# Prevalence and Determinants of Stunting-Anemia and Wasting-Anemia Comorbidities and Micronutrient Deficiencies in Children Under 5 in the Least-Developed Countries: A Systematic Review and Meta-analysis

**DOI:** 10.1093/nutrit/nuae063

**Published:** 2024-05-31

**Authors:** Getenet Dessie, Jinhu Li, Son Nghiem, Tinh Doan

**Affiliations:** College of Medicine and Health Science, Bahir Dar University, Bahir Dar, 79, Ethiopia, bduinfo@bdu.edu.et; Department of Health, Economics, Wellbeing and Society, National Centre for Epidemiology and Population Health (NCEPH), College of Health and Medicine, The Australian National University, Canberra, 2601, Australia, director.nceph@anu.edu.au; Department of Health, Economics, Wellbeing and Society, National Centre for Epidemiology and Population Health (NCEPH), College of Health and Medicine, The Australian National University, Canberra, 2601, Australia, director.nceph@anu.edu.au; Department of Health, Economics, Wellbeing and Society, National Centre for Epidemiology and Population Health (NCEPH), College of Health and Medicine, The Australian National University, Canberra, 2601, Australia, director.nceph@anu.edu.au; Department of Health, Economics, Wellbeing and Society, National Centre for Epidemiology and Population Health (NCEPH), College of Health and Medicine, The Australian National University, Canberra, 2601, Australia, director.nceph@anu.edu.au

## Abstract

**Context:**

Despite shifting from addressing isolated forms of malnutrition to recognizing its multifaceted nature, evidence on the prevalence and determinants of micronutrient deficiencies, and their coexistence with undernutrition in children under 5, remains insufficient, unsystematic, and incohesive.

**Objective:**

The aim of this systematic review and meta-analysis was to assess the prevalence and determinants of stunting-anemia and wasting-anemia comorbidities and micronutrient deficiencies in children under 5 in the least-developed countries (LDCs).

**Data Sources:**

Electronic searches took place from January 15, 2023, to February 14, 2024, across multiple databases, including PubMed, Embase, Web of Science, SCOPUS, African Index Medicus (AIM), World Health Organization's Institutional Repository for Information Sharing (IRIS), and African Journals Online. The search spanned the years 2000 to 2024, yet it yielded eligible full-text English research articles from only 2005 to 2021 conducted in LDCs. Studies lacking quantitative data on malnutrition types and their determinants were excluded.

**Data Extraction:**

Two independent authors assessed articles for bias and quality using Hoy et al's 10-item scale and Newcastle-Ottawa Scale (NOS) criteria. Prevalence and other details were extracted using a Joanna Briggs Institute Excel template. Authors extracted adjusted odds ratios (aORs) for determinant factors such as sex and vitamin A and iron supplementation.

**Data Analysis:**

The search yielded 6248 articles from 46 LDCs. Sixty-nine articles, with a total sample size of 181 605, met inclusion criteria for the final meta-analysis. Vitamin A deficiency affected 16.32% of children, and iodine deficiency affected 43.41% of children. The pooled prevalence of wasting-anemia and stunting-anemia comorbidity was 5.44% and 19.47%, respectively. Stunting was associated with vitamin A deficiency (aOR: 1.54; 95% CI: 1.01–2.37), and not taking vitamin A supplementation was associated with iron-deficiency anemia (aOR: 1.37; 95% CI: 1.21–1.55).

**Conclusion:**

A significant proportion of children under 5 in LDCs experienced stunting-anemia and wasting-anemia comorbidities and micronutrient deficiencies. This study underscores the urgent need to address factors driving these burdens.

**Systematic Review Registration:**

PROSPERO registration no. CRD42023409483.

## INTRODUCTION

Nutrition concerns have advanced beyond the existence of a single form of malnutrition, such as being underweight or obese, to now include the coexistence of multiple types of malnutrition, including the overlap of micronutrient deficiencies with other types of undernutrition. Over the past 20 years, evidence has indicated that undernutrition and overweight/obesity have increasingly co-occurred, posing a public health threat and affecting all sociodemographic and wealth/income categories.^1^^,^^2^ Hidden hunger, also known as micronutrient deficiency, has emerged as the third pillar in what has become a global issue, and is particularly challenging for low- and middle-income countries.^3^ Several pieces of evidence currently highlight the presence of a high burden of hidden hunger, in addition to undernutrition. Over 2 billion people worldwide experience hidden hunger, with over 1 billion of them living in developing countries.^3–5^

Multiple triggers, including poor maternal nutrition, nutrient-deficient diets for children, and a shift in the food system, contribute to the high burden of malnutrition encompassing both undernutrition and micronutrient deficiency.^6^ The accessibility of processed meals and drinks in low-income countries,^7^ as well as substantial declines in physical activity across all spheres of life because of the arrival of effort-saving devices, play significant roles in the emergence of new nutritional challenges.^8^ Least-developed countries (LDCs) are defined not just by low Gross National Income per capita but also by weak human assets (nutrition, health, education, adult literacy) and economic vulnerability (population size, remoteness, economic instability).^9^ Assessing malnutrition in these countries, particularly among children, is crucial for policy measures, because children in the least-developed nations are highly vulnerable to malnutrition due to a wide range of economic, social, and environmental factors.^10^

Owing to their geographical location and topography, the least-developed nations are undoubtedly vulnerable to various natural catastrophes, including recurring droughts and flooding during the rainy season, when most crops are produced.^11^^,^^12^ These countries also frequently encounter issues with food security and healthcare availability due to low investment in food production, conflict, and political unrest.^13–15^ This will increase the incidence of deficiency in micronutrients, important elements for human body functioning, which currently coexist with various forms of undernutrition in different populations. Despite the expected increase in the prevalence of these problems, there is limited evidence for LDCs. Regional reviews in sub-Saharan Africa^16^^,^^17^ have shown a high prevalence of undernutrition combined with overweight. However, undernutrition is a broad concept that includes stunting, wasting, and micronutrient deficiency. Unlike previous studies focused on the double burden of malnutrition, combining undernutrition with obesity/overweight at the household level, the current study delves into the simultaneous presence of different forms of undernutrition at the individual level, specifically targeting children under 5 years, a demographic where a significant gap in research is evident. This study also addresses underlying factors contributing to the coexistence of micronutrient deficiencies and other forms of undernutrition. In addition, the present study addresses significant public health issues, micronutrient deficiencies, and their determinants not covered in prior reviews, marking them as the primary focus of this study. The earlier studies^16^^,^^17^ also did not examine regional-level malnutrition prevalence to enable cross-region comparisons, an issue that has been addressed in the current study through region-specific subgroup analysis.

Given the existing research gaps and level of vulnerability, this study specifically focused on 3 critical malnutrition issues—hidden hunger (micronutrient deficiencies), stunting coexisting with anemia, and wasting coexisting with anemia—in LDCs. For micronutrient deficiencies, this study focuses on iron deficiency, vitamin A deficiency, and iodine deficiency due to their significant health impacts, public importance, and gaps in study. These deficiencies are particularly noteworthy for their profound and long-lasting health impacts on both individuals and communities. These 3 issues affect a large proportion of the population,^18^ especially pregnant women and children, leading to conditions such as blindness and impaired cognitive development and function, with iodine deficiency being the foremost cause of preventable intellectual disability in children worldwide.^19^^,^^20^ Additionally, malnourished children are deficient in many micronutrients, including vitamin A, iron, and iodine,^21^ making studying these micronutrients alongside other forms of undernutrition worthwhile. With regard to iron deficiency, the focus is on its severe form, iron-deficiency anemia. Due to its severity, iron-deficiency anemia is prioritized, emphasizing its importance, and advocating for widespread attention. Least-developed countries have implemented a significant national nutrition program^14^ across highly diverse geographic areas with a diverse range of socioeconomic groups. Addressing these issues in LDCs is vital. Therefore, this systematic review and meta-analysis was conducted to determine the pooled prevalence of the 3 types of malnutrition; micronutrient deficiencies, comorbidity of stunting with anemia, and comorbidity of wasting with anemia and their determinants, in children under 5 across LDCs to fill the existing knowledge gaps in the literature.

## METHODS

### Inclusion and exclusion criteria

All full-text studies conducted in LDCs between January 2000 and February 2024, and published in peer-reviewed journals or found in the gray literature, reporting the prevalence of iron-deficiency anemia, iodine deficiency, vitamin A deficiency, the coexistence of stunting with anemia, the coexistence of wasting with anemia, and any articles reporting factors for any of the above conditions among children under 5 were eligible for inclusion. The PICOS (Population, Intervention, Comparison, Outcomes, and Study design) details of the eligible studies are outlined in Table 1. With regard to language, all articles were initially downloaded without any language restrictions. However, due to practical constraints in translation, only articles in English were considered. Studies without quantitative data for any of the listed malnutrition types and their determinants were excluded.

**Tabel 1.** PICOS Criteria for Study Inclusion

Parameter	Criterion
Participants	Children under the age of 5
Interventions/exposure	Under-5 children with stunting who are not taking vitamin A supplementation, have not had antenatal care visits, do not consume vitamin A–rich fruits, vegetables, and meat, and are male
Comparisons	Children under 5 without stunting, who have received vitamin A supplementation, undergone antenatal care, consume vitamin A–rich fruits, vegetables, and meat, and are female
Outcomes	Coexistence of stunting with anemia and wasting with anemia and micronutrient deficiencies
Study design	Observational studies, including retrospective follow-up studies and laboratory-based cross-sectional studies

### Data sources

Electronic, web-based searches were conducted from January 15, 2023, to June 28, 2023, with updates made during revision (from January 22 to February 14, 2024) across multiple databases and search engines including PubMed, Google Scholar, Embase, Web of Science, SCOPUS, the African Index Medicus (AIM), the World Health Organization’s (WHO's) Institutional Repository for Information Sharing (IRIS), and African Journals Online were searched to find articles for this study. Additionally, further articles were accessed through searches of gray literature in institutional repositories and by reviewing reference lists from previously identified articles.

### Search strategy and selection criteria

This study follows the Preferred Reporting Items for Systematic Reviews and Meta-Analyses Protocols (PRISMA-P) checklist^22^ (see details in the S1 PRISMA checklist). The checklist uses all full-text articles published in peer-reviewed journals and found in the gray literature from 2000 to 2024 to synthesize plausible evidence from the available literature. The search strategy was constructed using terms based on the CoCoPop framework^23^—Co (Condition): “malnutrition,” “double burden,” “triple burden,” “coexisting,” “concurrent occurrence,” “micronutrient deficiencies,” “underweight,” “wasting,” “stunting,” “epidemiology,” “anemia,” “anaemia,” “iodine deficiency,” “Vitamin A deficiency,” “prevalence,” “associated factors,” “determinant factors”; Co (Context): “least developed countries”; and P (Population): “children,” “childhood”. Instead of using “LDCs,” the names of each of the 46 countries^24^^,^^25^ were combined with each condition and population. The search used the Boolean operator within each axis, combining key words with the “OR” operator, then linking the search strategies for the 2 axes with the “AND” operator (see details in S2 Searching strategy).

### Quality-assessment procedure

Predefined criteria such as being published in peer-reviewed journals or on websites of WHO, UNICEF, and institutional repositories; observational studies (eg, cross-sectional and longitudinal studies) published in English; conducted among children under 5; reporting the prevalence of iron-deficiency anemia, iodine deficiency, vitamin A deficiency, the coexistence of stunting with anemia, or the coexistence of wasting with anemia; and any articles reporting factors for any of the above problems were used to screen titles and abstracts for inclusion in the full-text evaluation. At this point, irrelevant articles were removed, and the full texts of the remaining articles were reviewed for inclusion in the final meta-analysis. The study used EndNote version 20 (Camelot UK Bidco Limited) to manage search results. Before the analysis, the Newcastle-Ottawa Scale (NOS) criteria^26^ were utilized to assess the quality of selected studies. Each article was critically examined by 2 independent reviewers using the NOS criteria. For gray literature, the first step was to establish inclusion criteria and decide which types of gray literature to consider. For example, the study did not include government reports and conference proceedings. Second, source credibility was assessed, with only credible sources like WHO, UNICEF websites, and institutional repositories included. Third, the quality and consistency of the presented data were evaluated. At the fourth stage, the methodological rigor of the studies was examined, involving the assessment of research design, sample size, statistical analysis, and the clarity of stated limitations. The authors also discussed including gray literature within the research team to establish a consensus based on collective judgment. After these processes, 2 authors critically and independently appraised each study. Disagreements among reviewers were resolved through discussion, involving a third reviewer if necessary. To determine whether an article should be included, the quality scores from the 2 independent reviewers were averaged. Articles with methodological flaws or unsatisfactory reporting of results were excluded from the final analysis.

### Data-abstraction procedure

Data from primary studies were extracted using an Excel spreadsheet (Microsoft Corporation, Redmond, WA, USA), developed in accordance with the Joanna Briggs Institute data-extraction form.^27^ The tool's suitability was tested using 5 studies. Specific details were collected from each study, which included the year of the study, geographic region, research design, sample size, diagnostic methods, the prevalence of each form of malnutrition (micronutrient deficiencies, stunting with anemia, and wasting with anemia), and the age range of the children studied, using a Microsoft Excel spreadsheet template. For independent variables, author information, year of the study, research design, sample size, adjusted odds ratio (aORs) of each study and their confidence interval (CI) were collected.

### Outcome measurement

This study comprehensively included 3 outcomes: the prevalence of coexistence of stunting with anemia, the prevalence of coexistence of wasting with anemia, and micronutrient deficiencies. The first goal of this review was to synthesize evidence by thoroughly examining the prevalence of 3 types of malnutrition—micronutrient deficiencies, the coexistence of stunting with anemia, and the coexistence of wasting with anemia—among children in LDCs in sub-Saharan Africa and other regions. The primary areas of focus for micronutrient deficiencies were iron-deficiency anemia, iodine deficiency, and vitamin A deficiency. The study's second goal was to conduct a literature review to identify determinant factors associated with micronutrient deficiencies, the coexistence of stunting with anemia, and the coexistence of wasting with anemia.

### Risk-of-bias assessment

To validate the study's methodological robustness and ensure reproducibility, Hoy et al's^28^ 10-item grading scale for prevalence studies was utilized to assess risk of bias in the included research. The evaluation included assessing the studies' sampling representativeness, randomness, nonresponse bias, the validity of the data-collection procedure, case definition, reliability and validity of study tool, and prevalence period. Each study was assigned a low or high risk of bias based on “yes” or “no” responses to domain questions. A score of 1 (yes) or 0 (no) was assigned to each domain for each study, and the sum of these domain values was used to generate the overall study quality score. Scores of 8–10, 6–7, and 0–5 indicate a “low,” “moderate,” and “high” risk of bias, respectively. Two independent reviewers assigned scores to each article, and disagreements among the reviewers were resolved through the involvement of a third reviewer and consensus.

### Statistical analysis

Further analysis was conducted using Stata (version 17; StataCorp, College Station, TX, USA). To assess heterogeneity across the studies, the inverse variance (*I^2^*) and Cochran's *Q* statistics were used.^29^ Given the substantial heterogeneity observed among the studies (*I^2^* > 25%), the pooled prevalence of micronutrient deficiencies, coexistence of stunting with anemia, and coexistence of wasting with anemia were computed using a random-effects model and reported with 95% CIs. Furthermore, a subgroup analysis was conducted based on geographic regions to investigate the distribution of micronutrient deficiencies, coexistence of stunting with anemia, and coexistence of wasting with anemia across subregions. A meta-regression analysis was also conducted to identify the source of observed heterogeneity in addition to subgroup analysis. Regression analysis was used to examine factors associated with micronutrient deficiencies and other comorbidities. Adjusted odds ratios were used to examine the relationship between identified variables and these malnutrition forms. The funnel plot asymmetry was used to subjectively detect publication bias and Egger's and Begg-Mazumdar rank correlation tests for objective evaluation.^30^ The leave-one-out sensitivity analysis was used to evaluate how a single study influenced the pooled estimates of vitamin A deficiency, iron-deficiency anemia, iodine deficiency, stunting-anemia comorbidity, and wasting-anemia comorbidity.

## RESULTS

### Search results

**Figure 1.**
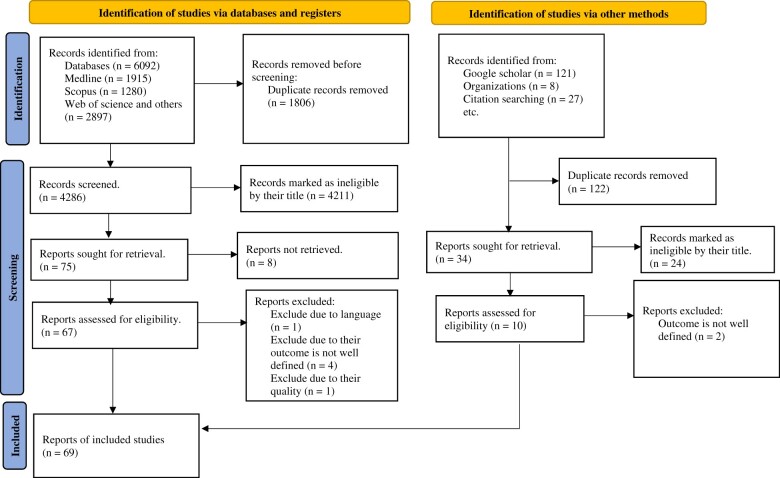
PRISMA (Preferred Reporting Items for Systematic Reviews and Meta-Analyses) Flow Diagram Showing the Procedure for Selecting Studies for Meta-analysis to Determine the Triple Burden of Malnutrition Among Children, 2000–2024, in Least-Developed Countries

The electronic search yielded 6248 articles: 6092 from databases and 156 from Google Scholar, organizations’ websites, and from citation searching. Among these, 1928 were identified as duplicates and subsequently excluded. Following a thorough examination of titles and abstracts, 4235 articles that were unrelated to the study were removed. Additionally, 8 articles were not retrieved because they were not freely available.^31–38^ After conducting a full-text review of the remaining 77 articles, 8 were excluded for various reasons: issues related to methodology, such as failing to report the sample size^39^; non–English-language articles^40^; and unclearly defined outcomes.^41–46^ Finally, 69 primary research articles were deemed eligible for inclusion in this systematic review. From these, 2 eligible articles^47^^,^^48^ were obtained to examine vitamin A deficiency, and another 2 articles^49^^,^^50^ were used to assess iodine deficiency, as the search strategy was updated during the revision process. However, they did not significantly change the pooled prevalence (Figure 1).

### Methodological quality of included studies

To assess the quality of the included studies, the NOS, modified for cross-sectional study design,^26^^,^^51^ was utilized. Among the 17 studies examining vitamin A deficiency, 1 study scored 6,^47^ 5 studies^41^^,^^52–56^ received a score of 8, signifying good quality, while the remaining studies scored within the range of 9 to 10, indicating very good quality.^48^^,^^51^^,^^57–65^ Among the 23 studies evaluating iron-deficiency anemia, 1 study^66^ was awarded a score of 7, 1 study^67^ scored 6, 9 studies^56^^,^^68–75^ scored 8, and the rest of the articles^59^^,^^64^^,^^65^^,^^76–84^ scored between 9 and 10. Among studies assessing iodine deficiency, 1 study^47^ scored 6, 2 studies^50^^,^^85^ scored 8, 1 study^49^ scored 9, and all other articles^86–88^ had a score of 10. With regard to studies investigating the concurrent presence of anemia with stunting and wasting, 2 studies received a score of 8,^89^^,^^90^ while the rest of the studies^72^^,^^91–113^ received a score of 10 (see details in S3 Quality assessment). Each study's methodological quality was also evaluated using Hoy et al's 10-item rating system for prevalence studies. According to the risk-of-bias assessment, 10 studies^57^^,^^66^^,^^98^^,^^100^^,^^106^^,^^112–116^ had a moderate risk of bias and 2 studies had a high risk of bias.^99^^,^^117^ The remaining studies had a low risk of bias (see details in S4 Risk bias assessment).

### Characteristics of the included studies

Three of the 17 research articles analyzed to determine the prevalence of vitamin A deficiency were from LDCs in Asia^47^^,^^48^^,^^62^ and the rest were from sub-Saharan Africa.^41^^,^^52–61^^,^^63–65^ Ethiopia^41^^,^^52^^,^^54^^,^^56–58^^,^^60^ ranked first in the number of all articles included. All of these articles were laboratory-based, cross-sectional studies. The included studies' sample sizes ranged from 204^57^ to 4589.^56^ Except for 4 articles^41^^,^^52^^,^^54^^,^^60^ that used clinical diagnosis, all others used laboratory methods, with 5 of them using high-performance liquid chromatography (HPLC) to identify vitamin A deficiency.^56–59^^,^^62^ All studies that used a laboratory approach to diagnose vitamin A deficiency used a cutoff point of 0.70 mol/L (see details in S5 Supportive Document, Tables S1–S4). This study also included 23 articles to assess iron-deficiency anemia: 91.3% were from sub-Saharan Africa,^59^^,^^64–69^^,^^71–74^^,^^76–84^^,^^118^ and the remaining 2 were from Asia.^70^^,^^75^ Individual studies had sample sizes ranging from 52^66^ to 24 348.^75^ The age of respondents ranged from 2 months to 60 months. Thirteen studies (56.52%) used the enzyme-linked immunosorbent assay (ELISA)^59^^,^^64^^,^^65^^,^^69^^,^^72^^,^^73^^,^^76–78^^,^^80^^,^^82^^,^^84^ to identify iron-deficiency anemia, while 6 studies^66^^,^^67^^,^^70^^,^^71^^,^^74^^,^^75^ did not specify the method used (see details in S5 Supportive document, Tables S1–S4).

Seven studies^47^^,^^49^^,^^50^^,^^85–88^ were conducted to assess the prevalence of iodine deficiency, with sample sizes ranging from 77^49^ to 1000.^47^ Four studies were conducted in sub-Saharan Africa,^49^^,^^50^^,^^85^^,^^86^ while the remaining 3 took place in Asia.^47^^,^^87^^,^^88^ The age of respondents varied from 6 to 60 months. Two studies were from Ethiopia,^85^^,^^86^ 2 from Cambodia,^47^^,^^88^ and the remaining studies were from Burkina Faso,^50^ Uganda,^49^ and Bangladesh.^87^ For diagnosing iodine deficiency, 2 studies used the Sandell-Kolthoff method,^49^^,^^88^ while the other 2 used ammonium persulfate methods.^50^^,^^85^ The diagnostic methods used in 1 study^47^ were not specified (see details in S5 Supportive document, Tables S1–S4).

Of all the articles used to assess the concurrent occurrence of anemia and malnutrition, 27 studies^72^^,^^89–113^^,^^119^ were included to assess the concomitant occurrence of anemia and stunting and 18 articles^72^^,^^81^^,^^92–98^^,^^101^^,^^102^^,^^105^^,^^106^^,^^109–112^ to assess the concurrent occurrence of anemia and wasting. Fourteen studies were from Ethiopia^89^^,^^90^^,^^93–95^^,^^97^^,^^100^^,^^102^^,^^104^^,^^109^^,^^111–113^^,^^119^ and 5 were from Bangladesh.^92^^,^^96^^,^^105^^,^^106^^,^^108^ All selected studies, which were cross-sectional, utilized WHO standards to diagnose malnutrition and anemia. The sample sizes of the individual studies ranged from 131^110^ to 8279.^111^ The age range of the respondents was 6 to 59 months, and all studies used a hemoglobin (Hb) level of 11.0 g/dL as the cutoff point to identify anemia in children (see details in S5 Supportive document, Tables S1–S4).

### Pooled prevalence of vitamin A deficiency

**Figure 2.**
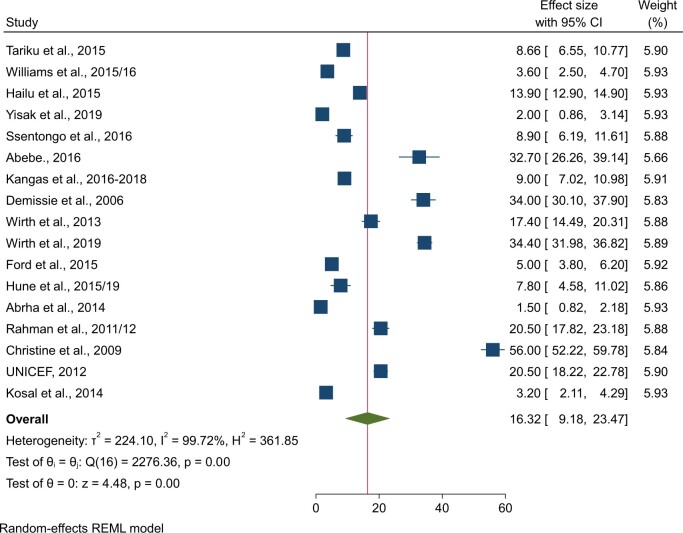
Forest Plot Showing the Pooled Prevalence of Vitamin A Deficiency Among Children Under 5: Least-Developed Countries, 2006–2019. *Abbreviation:* REML, Restricted Maximum Likelihood.

The pooled prevalence of vitamin A deficiency among 17 578 children in LDCs was 16.32% (95% CI: 9.18%, 23.47%; *I^2^* = 99.72%) (Figure 2). Due to apparent heterogeneity, a subgroup analysis was conducted based on the region where the studies were conducted. The prevalence of vitamin A deficiency among children in Asian LDCs was 14.68% (95% CI: 3.33%, 26.03%), in eastern Africa was 17.28% (95% CI: 7.40%, 27.17%), and in western Africa was 13.13% (95% CI: 4.90%, 21.36%) (Table 2).

**Table 2.** Subgroup Analyses of the Regional Distribution of Various Forms of Malnutrition Among Children Under 5 in Least-Developed Countries From 2005 to 2021

Problem type	Subgroup	No. of studies	Sample size, *n*	Pooled prevalence (95% CI)	Heterogeneity	
					*I^2^*	*P*
Vitamin A deficiency (subgrouped by regions)	LDCs in Asia	3	3073	14.68% (3.33%, 26.03%)	99%	<.01
	LDCs in eastern Africa	12	13 050	17.28% (7.40%, 27.17%)	99.81%	<.01
	LDCs in western Africa	2	1455	13.13% (4.90%, 21.36%)	95.44%	<.01
Iron-deficiency anemia (subgrouped by regions)	LDCs in Asia	2	24 679	15.92% (10.79%, 21.04%)	83.27%	<.01
	LDCs in eastern Africa	13	20 031	23.18% (15.74%, 30.62%)	99.32%	<.01
	LDCs in central Africa	4	3097	13.96% (2.81%, 25.11%)	98.98	<.01
	LDCs in northeast Africa	1	9703	24% (23.15%, 24.85%)	—	>.1
	LDCs in western Africa	3	2194	9.45% (0.24%, 19.15%)	98.77%	<.01
Iodine deficiency (subgrouped by regions)	LDCs in Asia	3	2345	56.23% (39.18%, 73.27%)	98.61%	<.01
	LDCs in Africa	4	2041	33.79% (1.07%, 68.67%)	99.80%	<.01
Comorbidity of stunting and anemia (subgrouped by regions)	LDCs in Asia	7	12 223	21.03% (18.44%, 23.61%)	89.46%	<.01
	LDCs in Caribbean	1	897	16.6% (14.17%, 19.03%)	—	
	LDCs in eastern Africa	18	39 945	18.81% (16.41%, 21.21%)	96.85%	<.01
	LDCs in western Africa	1	2524	23.7% (22.04%, 25.36%)	—	
Comorbidity of wasting and anemia (subgrouped by regions)	LDCs in Asia	6	9989	7.29% (4.93%, 9.66%)	93.57%	<.01
	LDCs in central Africa	1	432	7.64% (5.14%, 10.14%)	—	
	LDCs in eastern Africa	11	33 927	4.32% (2.80%, 5.85%)	97.93%	<.01

*Abbreviation:* LDC, least-developed country.

### Pooled prevalence of iron-deficiency anemia

**Figure 3.**
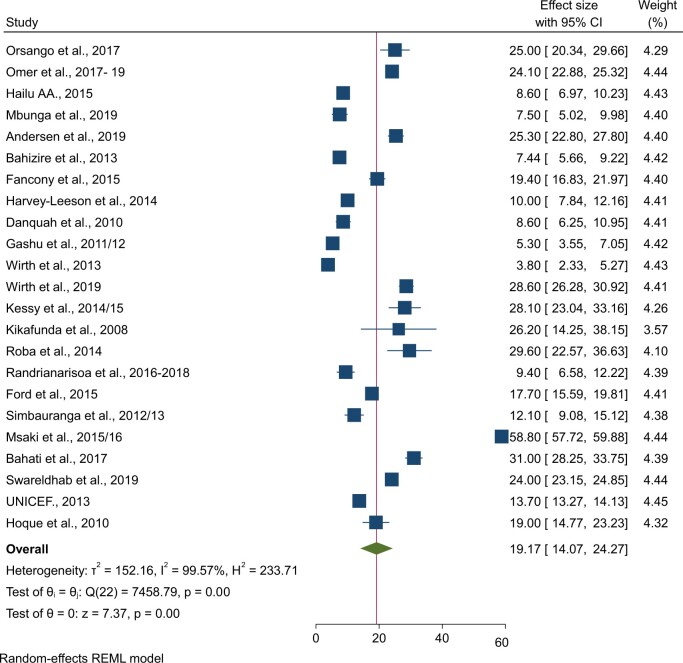
Forest Plot Showing the Pooled Prevalence of Iron Deficiency Anemia Among Children Under 5: Least-Developed Countries, 2008–2019

The pooled prevalence of iron-deficiency anemia among 59 704 children under 5 years in LDCs was 19.17% (95% CI: 14.07%, 24.27%; *I^2^* = 99.57%) (Figure 3). According to the subgroup analysis based on the region where the studies were conducted, northeast Africa and eastern Africa had the highest prevalence of iron-deficiency anemia among children under 5, with 24% (95% CI: 23.15%, 24.85%) in northeast Africa and 23.18% (95% CI: 15.74%, 30.62%) in eastern Africa, respectively (Table 2).

### Pooled prevalence of iodine deficiency

**Figure 4.**
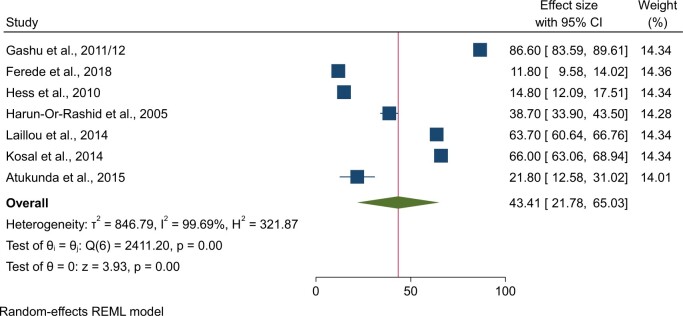
Forest plot Showing the Pooled Prevalence of Iodine Deficiency Among Under-5 Children: Least-Developed Countries, 2005–2018. *Abbreviation:* REML, Restricted Maximum Likelihood.

Seven studies were analyzed to estimate the pooled prevalence of iodine deficiency in 4386 children under 5 years in LDCs. The prevalence of iodine deficiency among children under 5 ranged from 11.8% to 86.6%. The pooled prevalence of iodine deficiency among children under 5 in LDCs was 43.41% (95% CI: 21.78%, 65.03%) (Figure 4). Regional subgroup analysis found that the least-developed nations in Asia had a more significant burden of iodine deficiency among children under 5, with 56.2% (95% CI: 39.2%, 73.27%), compared with the least-developed nations from sub-Saharan Africa, where the pooled prevalence in the region was 33.79% (95% CI: 1.09%, 68.67%) (Table 2).

### Pooled prevalence of comorbidity of stunting with anemia

**Figure 5.**
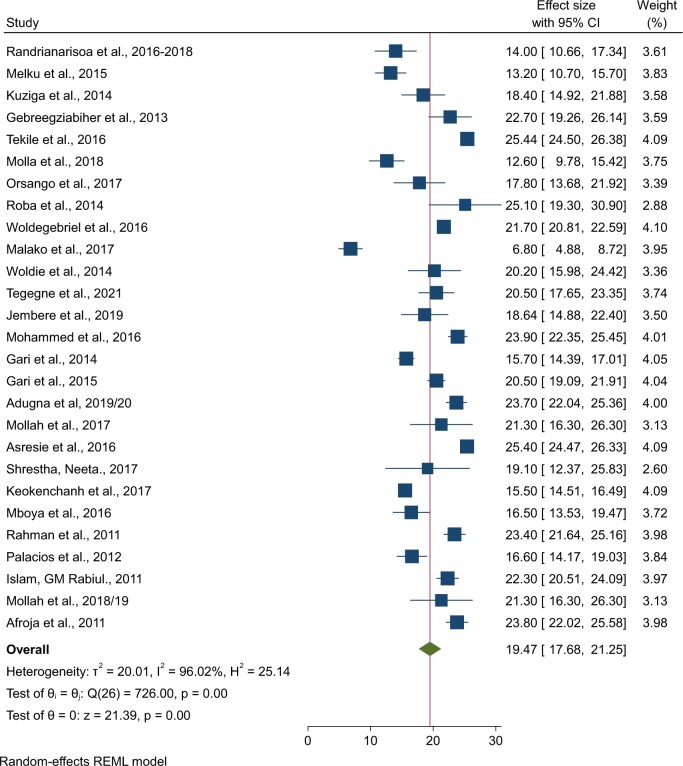
Forest Plot Showing the Pooled Prevalence of Stunting-Anemia Comorbidity Among Under-5 Children: Least-Developed Countries, 2011–2021. *Abbreviation:* REML, Restricted Maximum Likelihood.

Twenty-seven studies with a total sample size of 55 589 were included in the analysis to determine the pooled prevalence of stunting and anemia comorbidity. The prevalence of stunting-anemia comorbidity ranged from 6.8% to 25.44%. Based on the DerSimonian and Laird random-effects model, the pooled prevalence of stunting-anemia comorbidity was 19.47% (95% CI: 17.68%, 21.25%) (Figure 5). Least-developed countries in western Africa bear the highest burden of stunting-anemia comorbidity among under-5 children followed by LDCs in Asia, with a prevalence of 23.7% (95% CI: 22.04%, 25.36%) and 21.03% (95% CI: 18.44%, 23.61%), respectively. The pooled prevalences of comorbidity of stunting with anemia in LDCs in eastern Africa and the Caribbean region were 18.81% (95% CI: 16.41%, 21.21%) and 16.6% (95% CI: 14.17%, 19.03%), respectively (Table 2).

### Pooled prevalence of comorbidity of wasting with anemia

**Figure 6.**
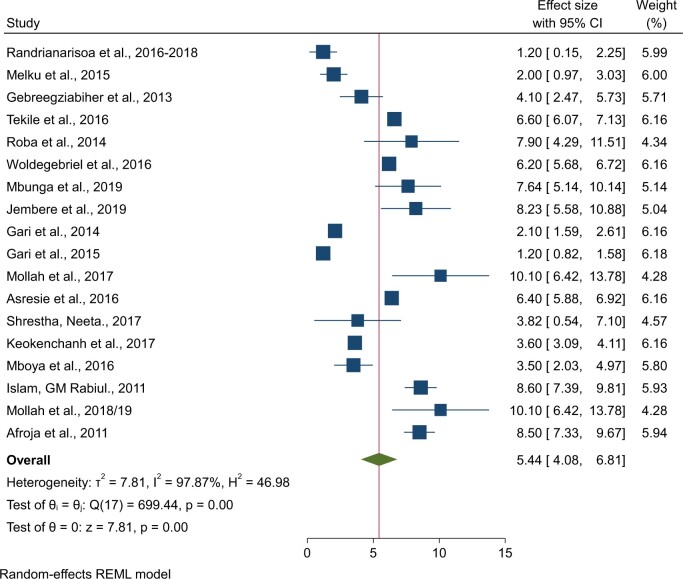
Forest Plot Showing the Pooled Prevalence of Wasting-Anemia Comorbidity Among Children Under 5: Least-Developed Countries, 2011–2019. *Abbreviation:* REML, Restricted Maximum Likelihood.

The analysis included 20 studies to evaluate the pooled prevalence of wasting-anemia comorbidity among 44 348 under-5 children. The pooled prevalence of wasting-anemia comorbidity was 5.44% (95% CI: 4.08%, 6.81%) (Figure 6). Central Africa and Asian LCDs had the highest prevalence of wasting-anemia comorbidity, with 7.64% (95% CI: 5.14%, 10.14%) and 7.3% (95% CI: 4.93%, 9.66%), respectively, compared with LDCs in eastern Africa, where the pooled prevalence was 4.32% (95% CI: 2.80%, 5.85%) (Table 2).

### Factors associated with vitamin A deficiency, iron deficiency, and stunting-anemia comorbidity

With regard to risk factors, children who had not received vitamin A supplementation were 1.5 times more likely to be deficient in vitamin A than children who had received vitamin A supplementation, with an aOR of 1.5 (95% CI: 1.0, 2.4). Concerning iron-deficiency anemia, stunting showed a significant association. Stunted children were 1.4 times more likely to have iron-deficiency anemia than non-stunted children, with an aOR of 1.4 (95% CI: 1.2, 1.6). A regression analysis was also conducted to investigate risk factors for anemia-stunting comorbidity. Although meta-regression analysis could not be performed due to a lack of articles, individual studies showed that consuming meat and taking iron supplements had a significant association with anemia-stunting comorbidity. Children who did not consume meat, did not consume vitamin A–rich fruits, and did not receive iron supplementation had a higher risk of anemia-stunting comorbidity, with an aOR of 1.5 (95% CI: 1.2, 2.1) and 2.9 (95% CI: 1.3, 6.2), respectively. Furthermore, children who did not consume vitamin A–rich fruits were 1.2 times more likely to develop anemia-stunting comorbidity than their counterparts, with an aOR of 1.2 (95% CI: 1.1, 1.3) (Table 3).

**Table 3.** Factors Associated With Vitamin A Deficiency, Iron Deficiency, and Stunting-Anemia Comorbidity in Least-Developed Countries, 2005–2021

Dependent variables	Independent variables	No. of articles included	aOR (95% CI)
Vitamin A deficiency	Vitamin A supplementation (reference, yes)^57^^,^^58^	2	1.5 (1.0, 2.4)*
	Stunting (reference, no)^57^^,^^63^	2	1.5 (1, 2.18)
	Antenatal care (reference, yes)^52^^,^^54^	2	2.7 (0.4, 16.7)
	Sex (reference, female)^41^^,^^52^^,^^58^^,^^60^	4	0.81 (0.3, 2.3)
Iron-deficiency anemia	Stunting (reference, no)^65^^,^^68^^,^^82^	3	1.4 (1.2, 1.6)*
	Sex (reference, female)^68^^,^^82^	2	1.5 (0.6, 2.4)
Anemia-stunting comorbidity	Sex (reference, female)^89^^,^^103^	2	2.1 (0.8, 5.9)
	No eating meat^103^	1	1.5 (1.2, 2.1)*
	No iron supplementation^89^	1	2.9 (1.3, 6.2)*
	Vitamin A–rich fruits and vegetables (reference, no)^103^	1	1.2 (1.1, 1.3)*

*Abbreviation:* aOR, adjusted odds ratio.

* Statistically significant: *P* < .05.

### Source of heterogeneity

Despite subgroup analysis, the results indicated the presence of heterogeneity across the studies. Therefore, a meta-regression analysis was conducted to identify the causes of this heterogeneity. The following study factors were included in the meta-regression analysis: year of study, child age, sample size, and region. The year of study was identified as a significant source of variation in the data used to determine vitamin A deficiency. Being in Asia's LDCs was also a significant source of heterogeneity for the data utilized to assess wasting and anemia comorbidity. However, none of the other variables were significant for the observed heterogeneity. In the context of meta-regression analysis for categorical variables such as “region,” the influence of individual dummy variables representing each subcategory within the “region” variable was initially examined. Subsequently, to assess the combined impact of these subcategories on the pooled effect's variability, a joint significance test was conducted after executing the meta-regression analysis. However, the aggregation of subcategories within the “region” variable did not show an overall significant effect on the pooled prevalence across all datasets (see details in S6 Source of heterogeneity).

### Publication bias

The subjective assessment of the funnel plot for all datasets revealed an uneven distribution of studies, suggesting potential publication bias (see details in S7 Meta funnel graphs). However, Begg’s intercept tests for all analyses indicated statistically significant publication bias in the included studies, as all *P*-values were greater than .05. In Egger's test, there was a significant publication bias in the datasets used to examine vitamin A deficiency and wasting anemia comorbidity, with β = 14.32 and a *P*-value <.01, and β = 2.71 with a *P*-value <.05, respectively. The meta-trim-and-fill analysis was conducted to examine the influence of publication bias on the pooled prevalence of vitamin A deficiency. Nevertheless, the effect size and CI remained similar, indicating no significant effect on the pooled analysis. Similarly, there was no significant difference in wasting-anemia comorbidity, with an observed prevalence of 5.44% (95% CI: 4.08, 6.81) compared to an observed + imputed prevalence of 5.24% (95% CI: 3.87, 6.61). There was no significant publication bias in Egger’s test for other datasets assessing iron-deficiency anemia, iodine deficiency, and stunting-anemia comorbidity, with β = 1.95, *P* = .387; β = −5.94, *P* = .56; and β = -0.34, *P* = .782, respectively.

### Sensitivity analysis

A leave-one-out sensitivity analysis also revealed that no single study significantly influenced the pooled estimate for vitamin A deficiency, iron-deficiency anemia, iodine deficiency, stunting-anemia comorbidity, and wasting-anemia comorbidity (see details in S8 Sensitivity analysis).

## DISCUSSION

This meta-analysis and systematic review aimed to synthesize evidence by thoroughly analyzing the extent of 3 types of malnutrition (micronutrient deficiencies, stunting-anemia comorbidity, and wasting-anemia comorbidity) among children under 5 years in LDCs. The study’s second goal was to identify determinant risk factors for micronutrient deficiencies, stunting-anemia comorbidity, and wasting-anemia comorbidity. The meta-analysis results revealed that the pooled prevalence of micronutrient deficiencies among children under the age of 5 was quite high in LDCs, ranging from 16.32% for vitamin A deficiency to 43.41% for iodine deficiency. Iron-deficiency anemia was also prevalent among children under 5 in LDCs, accounting for 19.47% of the total. This is similar to previous studies^120^^,^^121^ that show that, despite the worldwide decrease in micronutrient deficiencies, the prevalence of vitamin A and iron deficiency remains high in sub-Saharan Africa and South Asian countries.

The high degree of micronutrient deficiency could be attributed to a lack of a diversified and nutritious diet and food fortification and inadequate supplementation. Fortified and processed foods are now widely available to everyone in less-developed countries due to trade and globalization.^122^^,^^123^ While trade has undoubtedly improved food availability worldwide, it can also pose a threat to food security by increasing reliance on food imports, potentially making food supplies more insecure and endangering small farm holders’ competitiveness. Because most of today’s trading systems primarily focus on price and fiscal effectiveness, they might fail to include social and environmental impacts in market prices. This oversight can harm the environment and lead to less-healthy diets.^124^ Additionally, nations with lower Gross Domestic Product (GDP) per capita often have a diet high in micronutrient-deficient cereals or processed foods, which may lead to higher levels of micronutrient deficiency. Since cereals are less expensive than other food commodities, poorer households tend to consume more concentrated sources of energy that lack the dietary diversification required to meet their micronutrient requirements.^125^ On the other hand, fruits and vegetables, meat and dairy, legumes, seafood, nuts, and seeds are examples of micronutrient-rich foods that are not readily available in less-developed countries.^125^ The current study findings and other previous studies have significant policy implications for addressing micronutrient deficiency. In addition to promoting diverse food intake among the population, policymakers should pay particular attention to assessing the impact of trade policies and global market integration on a country’s food system. This study’s findings also underscore the need for micronutrient supplementation for children and support for diversified nutrition.

Despite the difference being nonsignificant, as indicated by the overlapping of CIs, iodine deficiency is more common than both vitamin A and iron deficiency. Previous comprehensive studies have also shown that iodine is the most common nutritional deficiency.^126^^,^^127^ This might be due to the fact that iodine deficiency is not easily preventable through increased dietary diversity.^128^ Despite efforts to expand household iodized salt consumption, it’s sustainability remains a challenge due to factors such as poverty, inadequate knowledge, negative perceptions of iodized salt, and poor practices in iodized salt consumption.^129^ Another explanation could be that universal salt iodization programs in some Asian countries rely on foreign financing, and both national governments and salt-producing groups have yet to develop organized implementation plans, affecting the programs' sustainability.^130^ Therefore, the high prevalence of iodine deficiency in both Asian and African LDCs highlights the need for interventions to promote sustainable iodized salt consumption and to address other key factors in both regions. One potential solution could be enhancing iodine intake by promoting the consumption of iodized salt.^126^ Additionally, increasing public awareness about iodized salt transportation, storage, and proper consumption practices, such as avoiding high-temperature cooking with iodized salt, could help reduce iodine deficiency.

Iron-deficiency anemia is more prevalent in least-developed northeast African countries than in the western Africa and Asia LDCs. This could be related to the persistent occurrence of extreme weather events and political crises in the Horn of Africa, such as drought, internal displacement due to conflict, and flooding.^131^ Sudan, the nation representing northeast Africa, has been at war for the past 3 decades, leading to the destruction of health institutions and the targeting of health workers.^132^ This may result in low community awareness about micronutrient consumption and a lack of service provision for micronutrient supply. All of these factors may contribute to an uneven diet and, ultimately, a high burden of iron-deficiency anemia. These findings suggest that, in addition to efforts aimed at promoting healthy dietary habits to prevent iron-deficiency anemia, policies should also address macrolevel and environmental conditions, such as mitigating political crises and addressing natural disasters.

Vitamin A deficiency was significantly associated with vitamin A supplementation. Although vitamin A is naturally present in many foods, individuals in developing nations often have limited access to vitamin A–rich foods. As a result, the WHO recommends that all children aged 6–59 months living in a community where vitamin A deficiency is a public health issue receive supplements.^133^ Therefore, the findings of this study emphasize the importance of strengthening the existing vitamin A supplementation program and increasing intake in children under the age of 5. Stunting was also found to have significant associations with iron-deficiency anemia. Stunting results from prolonged nutritional deficits, which can lead to low red blood cell counts and hemoglobin levels in the body, resulting in anemia, especially iron-deficiency anemia.^134^ Second, both anemia and stunting share similar disadvantageous sociodemographic characteristics, including poor household environment, low maternal education, poor living conditions, and low birth weight.^135^^,^^136^ This suggests the need for additional statistical analysis, such as bias analysis, to investigate the role of these factors further.

With regard to comorbidity, the combined prevalence of wasting anemia and stunting anemia was high. Anemia and undernutrition are both concentrated in socioeconomically disadvantaged groups, and they share numerous multifaceted causes involving complex interactions between diet, transmissible illnesses, and other factors, such as inadequate care and unhealthy household environments.^137^^,^^138^ The findings of these studies have implications that underscore the need to address anemia in children who are wasted and stunted. In addition to shared risk factors, there may be a biological relationship between undernutrition and anemia, which may warrant future investigation. In this review, factors such as meat consumption and the consumption of vitamin A–rich fruits and iron supplements were strongly linked to the comorbidity of anemia and stunting. All this evidence implies that any measures aimed at treating and reducing stunting should consider anemia.

In subgroup analysis, the LDCs in western Africa exhibited the highest burden of stunting and anemia comorbidity in comparison to the Caribbean and eastern Africa regions. This might be attributed to the fact that western African countries have the highest prevalence of anemia. A previous large-scale study^139^ found that the magnitude of anemia is higher in western Africa, primarily due to environmental and climatic factors. The health of vegetation and land surface temperature could play an important role in this variation. It is reasonable to assume that malnourished children are more likely to acquire anemia in areas with a high frequency of anemia, as revealed by a recent meta-analysis.^140^ On the other hand, due to the Caribbean's extensive coastlines and maritime culture, seafood is a fundamental part of the diet. This may afford the Caribbean population greater variety or access to micronutrient-rich foods.^141^ The current findings, along with those from previous studies, clearly demonstrate the need for customized interventions to address both malnutrition and anemia in those high-risk areas.

The current study exhibits heterogeneity. Heterogeneity is inevitable in meta-analysis due to differences in study quality, socioeconomic and geographical diversity, methodology, and participants. Consequently, this study used the random-effects model, which offers reliable estimation for heterogeneous studies.^142^^,^^143^ It is important to note that a high-level *I^2^* does not necessarily imply substantial variation, as it is widely known that heterogeneity tends to increase as the number of included studies increases.^144^ Therefore, exploring the origins of heterogeneity provides new insights into the study and sheds light on potential ways for future research. Since the analysis revealed the presence of high heterogeneity, various strategies were considered to mitigate it. First, subgroup analysis was conducted with several categorical variables and selected the results with the least heterogeneity for interpretation. However, persistent heterogeneity despite these efforts prompted further analysis using meta-regression to investigate various factors. These findings indicated that the year of the study and region were predictors of the observed heterogeneity.

The year of the study significantly contributed to the heterogeneity in data used to quantify vitamin A deficiency, indicating significant variations in the prevalence of vitamin A deficiency over different study years. This prompts an interesting research question: What factors drive these changes in micronutrient deficiencies over time? Additionally, what contributes to this temporal variation? An interesting research question, therefore, is what causes this change in micronutrient deficiencies over time and what factors contribute to this variation over time. Additionally, region was a significant source of heterogeneity in this meta-analysis used to estimate stunting-anemia comorbidity. In this analysis, the significance of region in assessing heterogeneity might be explained by the variation in the prevalence of the coexistence of anemia and stunting comorbidity between Asian LDCs and those in other continents.

### Limitations

An adequate number of articles in several regions could not be found, affecting the pooled prevalence for comparison. The current analysis found no articles on the comorbidity of overweight/obesity and micronutrient deficiency at the individual level, despite an expected high prevalence, which warrants future study.

## CONCLUSION

This meta-analysis found that a significant proportion of children under 5 years in LDCs worldwide have experienced comorbidities of stunting-anemia and wasting-anemia and micronutrient deficiencies. Western Africa exhibited higher rates of stunting-anemia comorbidity than eastern African and Caribbean LDCs. A high prevalence of iodine deficiency was observed in LDCs in Africa and Asia. Vitamin A supplementation has been linked to vitamin A deficiency, and stunting has been linked to iron-deficiency anemia. The consumption of meat and fruits rich in vitamin A and the intake of iron supplements were all significantly linked to the co-occurrence of anemia and stunting. The findings suggest that the burden of micronutrient deficiencies and wasting-anemia and stunting-anemia comorbidity among children has become an increasingly critical issue. Potential policy solutions could involve the establishment of national public health programs to promote salt iodization, as well as vitamin A and iron supplementation in the study region.

## Supplementary Material

nuae063_Supplementary_Data

## Data Availability

All data are available in the manuscript, and additional extracted data in Excel format will be shared upon reasonable request.
