# Supplementary material for: Prevalence and Determinants of Stunting-Anemia and Wasting-Anemia Comorbidities and Micronutrient Deficiencies in Children Under 5 in the Least-Developed Countries: A Systematic Review and Meta-analysis
Source: Nutr Rev. 2024 May 31;83(2):e178–94. doi: 10.1093/nutrit/nuae063 (PMC11723162; doi:10.1093/nutrit/nuae063)
Supplement: nuae063_Supplementary_Data [file nuae063_supplementary_data.zip › nuae063_Supplementary_Data/S2 Searching strategy.docx]

S2 Table **Search strategy of databases from January 1, 2023, to February 14, 2024.**

**For concurrent occurrence of malnutrition**

| **Medline** | | |
| --- | --- | --- |
| **No** | **Query** | **Records retrieved** |
| 1 | **Search:** **(triple burden) AND (Malnutrition)**  ("triple"[All Fields] OR "triples"[All Fields]) AND ("burden"[All Fields] OR "burdened"[All Fields] OR "burdening"[All Fields] OR "burdens"[All Fields]) AND ("malnutrition"[MeSH Terms] OR "malnutrition"[All Fields] OR "malnutrition s"[All Fields] OR "malnutrition"[All Fields] OR "malnutrition"[All Fields]) | 87 |
| 2 | **Search: (double burden) AND (Malnutrition)**  ("double"[All Fields] OR "doubled"[All Fields] OR "doubles"[All Fields] OR "doubling"[All Fields] OR "doublings"[All Fields]) AND ("burden"[All Fields] OR "burdened"[All Fields] OR "burdening"[All Fields] OR "burdens"[All Fields]) AND ("malnutrition"[MeSH Terms] OR "malnutrition"[All Fields] OR "malnutrition s"[All Fields] OR "malnutritional"[All Fields] OR "malnutritions"[All Fields]) | 772 |
| 3 | **Search: (coexisting) AND (Malnutrition)**  ("coexist"[All Fields] OR "coexistance"[All Fields] OR "coexistant"[All Fields] OR "coexisted"[All Fields] OR "coexistence"[All Fields] OR "coexistences"[All Fields] OR "coexistent"[All Fields] OR "coexisting"[All Fields] OR "coexists"[All Fields]) AND ("malnutrition"[MeSH Terms] OR "malnutrition"[All Fields] OR "malnutrition s"[All Fields] OR "malnutritional"[All Fields] OR "malnutritions"[All Fields]) | 1,232 |
| 4 | **Search:** **(concurrent occurrence) AND (Malnutrition)**  ("concurrent"[All Fields] OR "concurrently"[All Fields] OR "concurrents"[All Fields]) AND ("epidemiology"[MeSH Subheading] OR "epidemiology"[All Fields] OR "occurrence"[All Fields] OR "epidemiology"[MeSH Terms] OR "occurrences"[All Fields]) AND ("malnutrition"[MeSH Terms] OR "malnutrition"[All Fields] OR "malnutrition s"[All Fields] OR "malnutritional"[All Fields] OR "malnutritions"[All Fields]) | 402 |
| 5 | **Search: (micronutrient deficiencies) AND (underweight)**  ("micronutrients"[Pharmacological Action] OR "micronutrients"[MeSH Terms] OR "micronutrients"[All Fields] OR "micronutriments"[All Fields] OR "trace elements"[Pharmacological Action] OR "trace elements"[MeSH Terms] OR ("trace"[All Fields] AND "elements"[All Fields]) OR "trace elements"[All Fields] OR "micronutrient"[All Fields]) AND ("deficiencies"[All Fields] OR "deficiencies"[All Fields] OR "deficiency"[MeSH Subheading] OR "deficiency"[All Fields] OR "deficient"[All Fields] OR "deficients"[All Fields]) AND ("thinness"[MeSH Terms] OR "thinness"[All Fields] OR "underweight"[All Fields] OR "underweights"[All Fields]) | 513 |
| 6 | **Search: (micronutrient deficiencies) AND (Overweight)**  ("micronutrients"[Pharmacological Action] OR "micronutrients"[MeSH Terms] OR "micronutrients"[All Fields] OR "micronutriments"[All Fields] OR "trace elements"[Pharmacological Action] OR "trace elements"[MeSH Terms] OR ("trace"[All Fields] AND "elements"[All Fields]) OR "trace elements"[All Fields] OR "micronutrient"[All Fields]) AND ("deficiences"[All Fields] OR "deficiencies"[All Fields] OR "deficiency"[MeSH Subheading] OR "deficiency"[All Fields] OR "deficient"[All Fields] OR "deficients"[All Fields]) AND ("overweight"[MeSH Terms] OR "overweight"[All Fields] OR "overweighted"[All Fields] OR "overweightness"[All Fields] OR "overweights"[All Fields]) | 2613 |
| 7 | **Search: (((associated factors) OR (determinant factors)) OR (risk factors)) OR (predictors)**  (("associate"[All Fields] OR "associated"[All Fields] OR "associates"[All Fields] OR "associating"[All Fields] OR "association"[MeSH Terms] OR "association"[All Fields] OR "associations"[All Fields]) AND ("factor"[All Fields] OR "factor s"[All Fields] OR "factors"[All Fields])) OR (("analysis"[MeSH Subheading] OR "analysis"[All Fields] OR "determination"[All Fields] OR "determinant"[All Fields] OR "determinants"[All Fields] OR "determinate"[All Fields] OR "determinated"[All Fields] OR "determinates"[All Fields] OR "determinating"[All Fields] OR "determinations"[All Fields] OR "determine"[All Fields] OR "determined"[All Fields] OR "determines"[All Fields] OR "determining"[All Fields]) AND ("factor"[All Fields] OR "factor s"[All Fields] OR "factors"[All Fields])) OR ("risk factors"[MeSH Terms] OR ("risk"[All Fields] AND "factors"[All Fields]) OR "risk factors"[All Fields]) OR ("predictor"[All Fields] OR "predictors"[All Fields]) | **4,685,402** |
| 8 | Search: **under-five children**  "under-five"[All Fields] AND ("child"[MeSH Terms] OR "child"[All Fields] OR "children"[All Fields] OR "child s"[All Fields] OR "children s"[All Fields] OR "children’s"[All Fields] OR "child’s"[All Fields]) | 7312 |
| 9 | **Least developed countries*** | 14,696 |
| 10 | #6 AND #8 AND #9 AND #10 | 62 |
| 11 | #7 AND #8 AND #9 AND #10 | 31 |
| 12 | #1 AND #2 AND #3 OR #4 AND #5 AND #6 | 4,016 |
| 13 | #1 AND #2 AND #3 OR #4 AND #5 AND #6 AND #7 | 1,3011 |
| 14 | #1 AND #2 AND #3 OR #4 AND #5 AND #6 AND #7**AND #8 AND #9** | 22 |
| **Web of science** | | |
| **No** | **Query** | **Records retrieved** |
| 1 | **ALL= (triple burden) AND ALL=(Malnutrition)** | 123 |
| 2 | **ALL= (**Double **burden) AND ALL=(Malnutrition)** | 934 |
| 3 | **ALL= (coexisting) AND ALL=(Malnutrition)** | 267 |
| 4 | **ALL= (concurrent occurrence) AND ALL=(Malnutrition)** | 31 |
| 5 | **ALL= (micronutrient deficiencies) AND ALL=(Underweight)** | 241 |
| 6 | **ALL= (Micronutrient deficiencies) AND ALL=(Overweight)** | 455 |
| 7 | **ALL= (Associated factors) OR ALL= (Risk factors) OR ALL=(determinant factors) OR ALL=(Predictors )** | 4,917 |
| 8 | **ALL= (Under five children)** | 6322 |
| 9 | **Least developed countries*** | 23,256 |
| 10 | ALL= (micronutrient deficiencies AND underweight) AND ALL=(associated factors ) OR ALL=(Risk factors ) AND ALL=(determinant factors ) AND ALL=(predictors) AND ALL=(Under five children ) AND ALL=(least developed countries) | 45 |
| 11 | **ALL=(Overweight) AND ALL= (micronutrient deficiencies ) AND ALL=(associated factors ) OR ALL=(Risk factors ) AND ALL=(determinant factors ) AND ALL=(predictors) AND ALL=(Under five children ) AND ALL=(least developed countries)** | 76 |
| 12 | ALL=(triple burden of Malnutrition) AND ALL=(double burden Malnutrition) AND ALL=(coexisting malnutrition) OR TS=(concurrent occurrence and malnutrition) AND ALL=(micronutrient deficiencies AND underweight) AND ALL=(micronutrient deficiencies AND Overweight) AND ALL=(associated factors ) OR ALL=(Risk factors ) AND ALL=(determinant factors ) AND ALL=(predictors) AND ALL=(Under five children ) AND=ALL=(least developed countries) | 28 |
| 13 | **Limit 5 to English**(Languages), yr="2000 -Current" | 8 |
| **Scopus** | | |
| **No** | **Query** | **Records retrieved** |
| 1 | Triple AND burden AND malnutrition | 1,512 |
| 2 | Double AND burden AND malnutrition | 10,345 |
| 3 | Coexisting AND malnutrition | 1003 |
| 4 | concurrent AND occurrence AND malnutrition | 975 |
| 5 | Micronutrient AND deficiencies AND underweight | 2,514 |
| 6 | Micronutrient AND deficiencies AND overweight | 7,828 |
| 7 | Associated AND factors, AND risk AND factors AND predictors AND determinant AND factors | 251,712 |
| 8 | Under AND five AND children | 183,678 |
| 9 | **Least developed countries*** | 207,901 |
| 10 | triple AND burden AND malnutrition AND double AND burden AND malnutrition AND coexisting AND malnutrition OR concurrent AND occurrence AND malnutrition AND micronutrient AND deficiencies AND underweight AND overweight AND associated AND factors, AND risk AND factors AND predictors AND determinant AND factors AND under AND five AND children AND least developed countries * | 23 |
|  | Micronutrient AND deficiencies AND underweight AND associated AND factors, AND risk AND factors AND predictors AND determinant AND factors AND under AND five AND children AND least developed countries * | 345 |
| 3 | Micronutrient AND deficiencies AND overweight AND associated AND factors, AND risk AND factors AND predictors AND determinant AND factors AND under AND five AND children least developed countries * | 226 |
| **Instead of least developed countries*, the names of the countries listed below have been entered separately.**  *= (Angola OR Benin OR Burkina Faso OR Burundi OR Central African Republic OR Chad OR Comoros OR Democratic Republic of the Congo OR Djibouti OR Eritrea OR Ethiopia OR Gambia OR Guinea OR Guinea-Bissau OR Lesotho OR Liberia OR Madagascar OR Malawi OR Mali OR Mauritania OR Mozambique OR Niger OR Rwanda OR Sao Tome and Principe OR Senegal OR Sierra Leone OR Somalia OR South Sudan OR Sudan OR Togo OR Uganda OR United Republic of Tanzania OR Zambia OR Afghanistan OR Bangladesh OR Bhutan OR Cambodia OR Lao People’s Democratic Republic OR Myanmar OR Nepal OR Timor-Leste OR Yemen OR Haiti OR Kiribati OR Solomon Islands OR Tuvalu) | | |

**For Vitamin A deficiency**

| **Medline** | | |
| --- | --- | --- |
| **No** | **Query** | **Records retrieved** |
| 1 | **Search: Vitamin A deficiencies**  "Vitamin a deficiency"[MeSH Terms] OR "vitamin a deficiency"[All Fields] | 7,456 |
| 2 | **Search: (((associated factors) OR (determinant factors)) OR (risk factors)) OR (predictors)**  "risk factors"[MeSH Terms] OR ("risk"[All Fields] AND "factors"[All Fields]) OR "risk factors"[All Fields] OR (("associate"[All Fields] OR "associated"[All Fields] OR "associates"[All Fields] OR "associating"[All Fields] OR "association"[MeSH Terms] OR "association"[All Fields] OR "associations"[All Fields]) AND ("factor"[All Fields] OR "factor s"[All Fields] OR "factors"[All Fields])) OR "predictors"[All Fields] | 2,816,768 |
| 3 | **Search: under-five children**  "under"[All Fields] AND "five"[All Fields] AND ("child"[MeSH Terms] OR "child"[All Fields] OR "children"[All Fields] OR "child s"[All Fields] OR "children s"[All Fields] OR "childrens"[All Fields] OR "childs"[All Fields]) | 19671 |
| 4 | **Least developed countries*** | 14,831 |
| 5 | **#**1 AND **#**2 | 2,342,691 |
| 6 | **#1 AND #2 AND #3 AND #4** | 780 |
| **Web of science** | | |
| **No** | **Query** | **Records retrieved** |
| 1 | **ALL= (Vitamin A deficiency)** | 55,334 |
| 2 | **ALL= (Risk factors) OR ALL= (associated factors) OR ALL=(predictors)** | 2,952,456 |
| 3 | **ALL= (Under five children)** | 174,712 |
| 4 | **Least developed countries*** | 23,503 |
| 5 | **ALL= (Vitamin A deficiency) AND ALL= (associated factors ) OR ALL=(risk factors ) OR ALL=(predictors ) AND ALL=(under-five children)** | 1,551,011 |
| 6 | **Vitamin A deficiency (All Fields) and associated factors (All Fields) or Risk factors (All Fields) or predictors (All Fields) or determinant factors (All Fields) and under five children (All Fields) and least developed countries* (All Fields)** | 2345 |
| 6 | **Limit 5 to English**(Languages), yr="2000 -Current" | 1320 |
| **Scopus** | | |
| **No** | **Query** | **Records retrieved** |
| 1 | vitamin AND A AND deficiency | 132,712 |
| 2 | Associated AND factors, AND risk AND factors AND predictors | 1,100,915 |
|  | under AND five AND children | 183,711 |
| 4 | **Least developed countries*** | 207,912 |
| 5 | **vitamin AND a AND deficiency AND associated AND factors OR risk AND factors OR predictors AND among AND under-five AND children AND Least developed countries*** | 865 |
| **Instead of least developed countries*, the names of the countries listed below have been entered separately.**  *= ((Angola OR Benin OR Burkina Faso OR Burundi OR Central African Republic OR Chad OR Comoros OR Democratic Republic of the Congo OR Djibouti OR Eritrea OR Ethiopia OR Gambia OR Guinea OR Guinea-Bissau OR Lesotho OR Liberia OR Madagascar OR Malawi OR Mali OR Mauritania OR Mozambique OR Niger OR Rwanda OR Sao Tome and Principe OR Senegal OR Sierra Leone OR Somalia OR South Sudan OR Sudan OR Togo OR Uganda OR United Republic of Tanzania OR Zambia OR Afghanistan OR Bangladesh OR Bhutan OR Cambodia OR Lao People’s Democratic Republic OR Myanmar OR Nepal OR Timor-Leste OR Yemen OR Haiti OR Kiribati OR Solomon Islands OR Tuvalu)) | | |

**For Iron deficiency anaemia**

| **Medline** | | |
| --- | --- | --- |
| **No** | **Query** | **Records retrieved** |
| 1 | **Search: Iron deficiency anaemia**  "Iron deficiency anaemia"[All Fields] OR "anemia, iron deficiency"[MeSH Terms] OR ("anemia"[All Fields] AND "iron deficiency"[All Fields]) OR "iron-deficiency anemia"[All Fields] OR ("iron"[All Fields] AND "deficiency"[All Fields] AND "anemia"[All Fields]) OR "iron deficiency anemia"[All Fields] | **23,423** |
| 2 | **Search: (((associated factors) OR (determinant factors)) OR (risk factors)) OR (predictors)**  "risk factors"[MeSH Terms] OR ("risk"[All Fields] AND "factors"[All Fields]) OR "risk factors"[All Fields] OR (("associate"[All Fields] OR "associated"[All Fields] OR "associates"[All Fields] OR "associating"[All Fields] OR "association"[MeSH Terms] OR "association"[All Fields] OR "associations"[All Fields]) AND ("factor"[All Fields] OR "factor s"[All Fields] OR "factors"[All Fields])) OR "predictors"[All Fields] | **2,985,121** |
| 3 | **Search: under-five children**  "child"[MeSH Terms] OR "child"[All Fields] OR "children"[All Fields] OR "child's"[All Fields] OR "children's"[All Fields] OR "children"[All Fields] OR "childs"[All Fields] | **20149** |
| 4 | **Least developed countries*** | **14,687** |
| 5 | **#**1 AND **#**2 | **3565** |
| 6 | **#**1 AND **#**2 AND **#**3 AND **#**4 | **671** |
| **Web of science** | | |
| **No** | **Query** | **Records retrieved** |
| 1 | **ALL= (Iron deficiency anaemia)** | 22,367 |
| 2 | **ALL= (associated factors) OR ALL= (Risk factors) OR ALL= (determinant factors) OR ALL= (Predictors)** | 1,597,211 |
| 3 | **ALL= (Under five children)** | 17,698 |
| 4 | **Least developed countries*** | 37,127 |
| 5 | ALL= (Under five children) AND ALL= (Iron deficiency anaemia) | 132 |
| 5 | **ALL= (Iron deficiency anaemia) AND ALL= (associated factors) OR ALL= (Risk factors) OR ALL=(predictors ) AND ALL=(Under five children )** | 1,569 |
| 6 | **ALL= (Iron deficiency anaemia) AND ALL= (associated factors) OR ALL= (Risk factors) OR ALL= (predictors) AND ALL= (Under five children) AND other least developed countries* (All Fields)** | 1,553 |
| 6 | **Limit 5 to English**(Languages), yr="2000 -Current" | 959 |
| **Scopus** | | |
| **No** | **Query** | **Records retrieved** |
| 1 | Iron deficiency anaemia | 101,373 |
| 2 | associated AND factors, AND risk AND factors AND predictors | 1,100,844 |
| 3 | under AND five AND children | 29,197 |
| 4 | **Least developed countries*** | 207,776 |
| 5 | **Iron AND deficiency AND anaemia AND associated AND factors, AND risk AND factors AND predictors AND under-five AND Least developed AND countries*** | 155 |
| **Instead of least developed countries*, the names of the countries listed below have been entered separately.**  *= (Angola OR Benin OR Burkina Faso OR Burundi OR Central African Republic OR Chad OR Comoros OR Democratic Republic of the Congo OR Djibouti OR Eritrea OR Ethiopia OR Gambia OR Guinea OR Guinea-Bissau OR Lesotho OR Liberia OR Madagascar OR Malawi OR Mali OR Mauritania OR Mozambique OR Niger OR Rwanda OR Sao Tome and Principe OR Senegal OR Sierra Leone OR Somalia OR South Sudan OR Sudan OR Togo OR Uganda OR United Republic of Tanzania OR Zambia OR Afghanistan OR Bangladesh OR Bhutan OR Cambodia OR Lao People’s Democratic Republic OR Myanmar OR Nepal OR Timor-Leste OR Yemen OR Haiti OR Kiribati OR Solomon Islands OR Tuvalu) | | |

**For iodine deficiency**

| **Medline** | | |
| --- | --- | --- |
| **No** | **Query** | **Records retrieved** |
| 1 | **Search: iodine deficiency**  ("halogenation"[MeSH Terms] OR "halogenation"[All Fields] OR "iodination"[All Fields] OR "iodin"[All Fields] OR "iodinate"[All Fields] OR "iodinated"[All Fields] OR "iodinates"[All Fields] OR "iodinating"[All Fields] OR "iodinations"[All Fields] OR "iodine"[MeSH Terms] OR "iodine"[All Fields] OR "iodines"[All Fields]) AND ("deficiencies"[All Fields] OR "deficiencies"[All Fields] OR "deficiency"[MeSH Subheading] OR "deficiency"[All Fields] OR "deficient"[All Fields] OR "deficient"[All Fields]) | **7,988** |
| 2 | **Search: (((associated factors) OR (determinant factors)) OR (risk factors)) OR (predictors)**  (("associate"[All Fields] OR "associated"[All Fields] OR "associates"[All Fields] OR "associating"[All Fields] OR "association"[MeSH Terms] OR "association"[All Fields] OR "associations"[All Fields]) AND ("factor"[All Fields] OR "factor s"[All Fields] OR "factors"[All Fields])) OR ("risk factors"[MeSH Terms] OR ("risk"[All Fields] AND "factors"[All Fields]) OR "risk factors"[All Fields]) OR (("analysis"[MeSH Subheading] OR "analysis"[All Fields] OR "determination"[All Fields] OR "determinant"[All Fields] OR "determinants"[All Fields] OR "determinate"[All Fields] OR "determinated"[All Fields] OR "determinates"[All Fields] OR "determinating"[All Fields] OR "determinations"[All Fields] OR "determine"[All Fields] OR "determined"[All Fields] OR "determines"[All Fields] OR "determining"[All Fields]) AND ("factor"[All Fields] OR "factor s"[All Fields] OR "factors"[All Fields])) | **4,422,195** |
| 3 | **Search: under-five children**  "under-five"[All Fields] AND ("child"[MeSH Terms] OR "child"[All Fields] OR "children"[All Fields] OR "child s"[All Fields] OR "children s"[All Fields] OR "childrens"[All Fields] OR "childs"[All Fields]) | **6,217** |
| 4 | **Least developed countries*** | **14,701** |
| 5 | **#**1 AND #2 | 1,701 |
| 6 | **#**1 AND **#**2 AND **#**3 AND **#**4 | **422** |
| **Web of science** | | |
| **No** | **Query** | **Records retrieved** |
| 1 | **ALL= (iodine deficiency)** | 6,217 |
| 2 | **ALL= (associated factors) OR ALL= (Risk factors) OR ALL=(determinant factors) OR ALL=(Predictors )** | 3,028,534 |
| 3 | **ALL= (Under five children)** | 5,861 |
| 4 | Least developed countries * | 25,132 |
| 5 | ALL= (iodine deficiency) AND ALL= (Under five children) | 34 |
| 5 | **ALL= (iodine deficiency) AND ALL= (associated factors) OR ALL=(Risk factors ) OR ALL=(determinant factors) OR ALL=(predictors ) AND ALL=(Under five children )** | 187 |
| 6 | **ALL= (iodine deficiency) AND ALL= (associated factors) OR ALL= (Risk factors) OR ALL= (determinant factors) OR ALL= (predictors) AND ALL= (Under five children) AND**  **ALL= (least developed countries*)** | 556 |
| 6 | **Limit 5 to English**(Languages), yr="2000 -Current" | 532 |
| **Scopus** | | |
| **No** | **Query** | **Records retrieved** |
| 1 | Iodine deficiency | 42,079 |
| 2 | associated AND factors, AND risk AND factors AND predictors AND **determinant AND factors** | 251,489 |
| 3 | under AND five AND children | 59,254 |
| 4 | **Least developed countries*** | 207,798 |
| 5 | **Iodine AND deficiency AND associated AND factors, AND risk AND factors AND predictors AND determinant AND factors AND under-five AND** least developed **AND countries*** | 34 |
| **Instead of least developed countries*, the names of the countries listed below have been entered separately.**  *= (Angola OR Benin OR Burkina Faso OR Burundi OR Central African Republic OR Chad OR Comoros OR Democratic Republic of the Congo OR Djibouti OR Eritrea OR Ethiopia OR Gambia OR Guinea OR Guinea-Bissau OR Lesotho OR Liberia OR Madagascar OR Malawi OR Mali OR Mauritania OR Mozambique OR Niger OR Rwanda OR Sao Tome and Principe OR Senegal OR Sierra Leone OR Somalia OR South Sudan OR Sudan OR Togo OR Uganda OR United Republic of Tanzania OR Zambia OR Afghanistan OR Bangladesh OR Bhutan OR Cambodia OR Lao People’s Democratic Republic OR Myanmar OR Nepal OR Timor-Leste OR Yemen OR Haiti OR Kiribati OR Solomon Islands OR Tuvalu) | | |
