# Supplementary material for: Prevalence and Determinants of Stunting-Anemia and Wasting-Anemia Comorbidities and Micronutrient Deficiencies in Children Under 5 in the Least-Developed Countries: A Systematic Review and Meta-analysis
Source: Nutr Rev. 2024 May 31;83(2):e178–94. doi: 10.1093/nutrit/nuae063 (PMC11723162; doi:10.1093/nutrit/nuae063)
Supplement: nuae063_Supplementary_Data [file nuae063_supplementary_data.zip › nuae063_Supplementary_Data/S3 Quality assessment.docx]

**Newcastle-Ottawa Scale adapted for cross-sectional studies**

**Selection:**

**1. Representativeness of the sample:**

a. Truly representative of the average in the target population. * (all subjects or random sampling)

b. Somewhat representative of the average in the target group. * (non-random sampling)

c. Selected group of users/convenience sample.

d. No description of the derivation of the included subjects.

**2. Sample size:**

a. Justified and satisfactory (including sample size calculation). *

b. Not justified.

c. No information provided

**3. Non-respondents:**

a. Proportion of target sample recruited attains pre-specified target or basic summary of non-respondent characteristics in sampling frame recorded. *

b. Unsatisfactory recruitment rate, no summary data on non-respondents.

c. No information provided

**4. Ascertainment of the exposure (risk factor):**

a. Vaccine records/vaccine registry/clinic registers/hospital records only. **

b. Parental or personal recall and vaccine/hospital records. *

c. Parental/personal recall only.

**Comparability: (Maximum 2 stars)**

**1. Comparability of subjects in different outcome groups on the basis of design or analysis. Confounding factors controlled.**

a. Data/ results adjusted for relevant predictors/risk factors/confounders e.g. age, sex, time since vaccination, etc. **

b. Data/results not adjusted for all relevant confounders/risk factors/information not provided.

**Outcome:**

**1. Assessment of outcome:**

a. Independent blind assessment using objective validated laboratory methods. **

b. Unblended assessment using objective validated laboratory methods. **

c. Used non-standard or non-validated laboratory methods with gold standard. *

d. No description/non-standard laboratory methods used.

**2. Statistical test:**

a. Statistical test used to analyse the data clearly described, appropriate and measures of association presented including confidence intervals and probability level (p value). *

b. Statistical test not appropriate, not described or incomplete.

**Cross-sectional Studies:**

**Very Good Studies: 9-10 points**

Good Studies: 7-8 points

Satisfactory Studies: 5-6 points

Unsatisfactory Studies: 0 to 4 points

This scale has been adapted from the Newcastle-Ottawa Quality Assessment Scale for cohort studies to provide quality assessment of cross sectional studies^12^.

*1. Herzog R, Álvarez-Pasquin M, Díaz C, Del Barrio JL, Estrada JM, Gil Á. Are healthcare workers’ intentions to vaccinate related to their knowledge, beliefs and attitudes? A systematic review. BMC public health. 2013 Dec;13(1):1-7.*

*2. Stang A: Critical evaluation of the Newcastle-Ottawa scale for the assessment of the quality of nonrandomized studies in meta-analyses. European journal of epidemiology 2010, 25(9):603-605.*

**Assessment Result**s

**Note that**

- **No star**

*** 1 point**

**** 2 points**

**Quality assessment for articles to assess vitamin A deficiency**

| Id | Studies | Representativeness of the sample (*) | Sample size (*) | Non-respondents (*) | Ascertainment of the exposure (**, *) | Comparability  (**) | Assessment of the outcome (**, *) | Statistical test  (*) | Total |
| --- | --- | --- | --- | --- | --- | --- | --- | --- | --- |
| 1 | Tariku et al/2015. | * | * | * | ** | ** | - | * | 8 |
| 2 | Williams et al./2015/16. | * | * | * | ** | - | ** | * | 8 |
| 3 | Hailu et al/2015. | * | * | * | ** | - | ** | * | 8 |
| 4 | Ssentongo et al/2016. | * | * | * | ** | ** | ** | * | 10 |
| 5 | Abebe /2016. | * | * | * | ** | ** | ** | * | 10 |
| 6 | Kangas et al /2016-2018. | * | * | * | ** | ** | ** | * | 10 |
| 7 | Demissie et al/2006. | * | * | * | ** | ** | ** | * | 9 |
| 8 | Wirth et al/2013. | * | * | * | ** | ** | ** | * | 10 |
| 9 | Wirth et al/2019. | * | * | * | ** | ** | ** | * | 10 |
| 10 | Ford et al/2015. | * | * | * | ** | ** | ** | * | 10 |
| 11 | Hune et al/2015-19. | * | * | * | ** | ** | ** | * | 10 |
| 12 | Rahman et al/2011/12. | * | * | * | ** | ** | ** | * | 10 |
| 13 | Yisak et al/2019. | * | * | * | ** | ** | - | * | 8 |
| 14 | Abrha et al/2014. | * | * | * | ** | ** | - | * | 8 |
| 15 | Christine et al/2009. | * | * | * | ** | ** | - | * | 8 |
| 16 | UNICEF/2012 | * | * | * | ** | ** | ** | * | 10 |
| 17 | Kosal et al/2014. | * | * | * | ** | - | - | * | 6 |

**Quality assessment for articles to assess iron deficiency anemia**

| Id | Studies | Representativeness of the sample (*) | Sample size (*) | Non-respondents (*) | Ascertainment of the exposure (**, *) | Comparability  (**) | Assessment of the outcome (**, *) | Statistical test  (*) | Total |
| --- | --- | --- | --- | --- | --- | --- | --- | --- | --- |
| 1 | Orsango et al/2017. | * | * | * | ** | ** | - | * | 8 |
| 2 | Omer et al/2017-19. | * | - | * | ** | ** | ** | * | 9 |
| 3 | Hailu AA/2015. | * | * | * | ** | - | ** | * | 8 |
| 4 | Mbunga et al/2019. | * | * | * | ** | ** | ** | * | 10 |
| 5 | Andersen et al/2019. | * | * | * | ** | - | - | * | 6 |
| 6 | Bahizire et al/2013. | * | * | * | ** | ** | ** | * | 10 |
| 7 | Fançony et al/2015. | - | - | * | ** | ** | ** | * | 8 |
| 8 | Harvey-Leeson et al/2014. | * | * | * | - | ** | ** | * | 8 |
| 9 | Danquah et al/2010. | * | * | * | ** | ** | ** | * | 10 |
| 10 | Gashu et al/2011/12. | * | * | * | ** | ** | ** | * | 10 |
| 11 | Wirth et al/2013. | * | * | * | ** | ** | ** | * | 10 |
| 12 | Wirth et al/2019. | * | * | * | ** | ** | ** | * | 10 |
| 13 | Kessy et al/2014/15. | * | * | * | ** | ** | ** | * | 10 |
| 14 | Kikafunda et al/2008. | * | - | * | ** | ** | - | * | 7 |
| 15 | Roba et al/2014. | - | - | * | ** | ** | ** | * | 8 |
| 16 | Randrianarisoa et al/2016-2018. | - | - | * | ** | ** | ** | * | 8 |
| 17 | Ford et al/2015. | * | * | * | ** | ** | ** | * | 10 |
| 18 | Simbauranga et al/2012/13. | * | * | * | ** | ** | ** | * | 10 |
| 19 | Msaki et al/2015/16. | * | * | * | ** | ** | ** | * | 10 |
| 20 | Bahati et al/2017. | * | * | * | ** | ** | ** | * | 10 |
| 21 | Swareldhab et al/2019. | * | * | * | ** | ** | - | * | 8 |
| 22 | UNICEF/2013. | * | * | * | ** | ** | - | * | 8 |
| 23 | Hoque et al/2010. | * | * | * | ** | ** | - | * | 8 |

**Quality assessment for articles to assess iodine deficiency.**

| Id | Studies | Representativeness of the sample (*) | Sample size (*) | Non-respondents (*) | Ascertainment of the exposure (**, *) | Comparability  (**) | Assessment of the outcome (**, *) | Statistical test  (*) | Total |
| --- | --- | --- | --- | --- | --- | --- | --- | --- | --- |
| 1 | Gashu et al/2011/12. | * | * | * | ** | ** | ** | * | 10 |
| 2 | Ferede et al/2018. | * | * | * | ** | ** | - | * | 8 |
| 3 | Hess et al/2010. | * | * | * | ** | - | ** | * | 8 |
| 4 | Harun-Or-Rashid et al/2005. | * | * | * | ** | ** | ** | * | 10 |
| 5 | Laillou et al/2014. | * | * | * | ** | ** | ** | * | 10 |
| 6 | Kosal et al/2014. | * | * | * | ** | - | - | * | 6 |
| 7 | Atukunda et al/2015. | * | - | * | ** | ** | ** | * | 9 |

**Quality assessment for articles to assess concurrent occurrence of anemia with wasting or stunting.**

| Id | Studies | Representativeness of the sample (*) | Sample size (*) | Non-respondents (*) | Ascertainment of the exposure (**, *) | Comparability  (**) | Assessment of the outcome (**, *) | Statistical test  (*) | Total |
| --- | --- | --- | --- | --- | --- | --- | --- | --- | --- |
| 1 | Randrianarisoa et al/2016-18. | * | * | * | ** | ** | ** | * | 10 |
| 2 | Melku et al/2015. | * | * | * | ** | ** | ** | * | 10 |
| 3 | Kuziga et al/2014. | * | * | * | ** | ** | ** | * | 10 |
| 4 | Gebreegziabiher et al/2013. | * | * | * | ** | ** | ** | * | 10 |
| 5 | Tekile et al/2016. | * | * | * | ** | ** | ** | * | 10 |
| 6 | Molla et al/2018. | * | * | * | ** | ** | ** | * | 10 |
| 7 | Orsango et al/2017. | * | * | * | ** | ** | ** | * | 8 |
| 8 | Roba et al/2014. | * | * | * | ** | ** | ** | * | 10 |
| 9 | Woldegebriel et al/2016. | * | * | * | ** | ** | ** | * | 10 |
| 10 | Malako et al/2017. | * | * | * | ** | ** | ** | * | 10 |
| 11 | Woldie et al/2014. | * | * | * | ** | ** | ** | * | 10 |
| 12 | Tegegne et al/2021. | * | * | * | ** | ** | ** | * | 8 |
| 13 | Jembere et al/2019. | * | * | * | ** | ** | ** | * | 10 |
| 14 | Mohammed et al/2016. | * | * | * | ** | ** | ** | * | 10 |
| 15 | Gari et al/2014. | * | * | * | ** | ** | ** | * | 10 |
| 16 | Gari et al/2015. | * | * | * | ** | ** | ** | * | 10 |
| 17 | Adugna et al/2019/20 | * | * | * | ** | ** | ** | * | 10 |
| 18 | Mollah et al/2017. | * | * | * | ** | ** | ** | * | 10 |
| 19 | Asresie et al/2016. | * | * | * | ** | ** | ** | * | 10 |
| 20 | Shrestha, Neeta/2017. | * | * | * | ** | ** | ** | * | 10 |
| 21 | Keokenchanh et al/2017. | * | * | * | ** | ** | ** | * | 10 |
| 22 | Mboya et al/2016. | * | * | * | ** | ** | ** | * | 10 |
| 23 | Rahman et al/2011. | * | * | * | ** | ** | ** | * | 10 |
| 24 | Palacios et al/2012. | * | * | * | ** | ** | ** | * | 10 |
| 25 | Islam, GM Rabiul/2011. | * | * | * | ** | ** | ** | * | 10 |
| 26 | Mollah et al/2018/19. | * | * | * | ** | ** | ** | * | 10 |
| 27 | Afroja et al/2011. | * | * | * | ** | ** | ** | * | 10 |
