# Supplementary material for: Prevalence and Determinants of Stunting-Anemia and Wasting-Anemia Comorbidities and Micronutrient Deficiencies in Children Under 5 in the Least-Developed Countries: A Systematic Review and Meta-analysis
Source: Nutr Rev. 2024 May 31;83(2):e178–94. doi: 10.1093/nutrit/nuae063 (PMC11723162; doi:10.1093/nutrit/nuae063)
Supplement: nuae063_Supplementary_Data [file nuae063_supplementary_data.zip › nuae063_Supplementary_Data/S4 risk bias assessment.pdf]

Table S4: Risk of Bias assessment Tool of Eligible Articles by using the Hoy 2012 tool

NO	Study ID	Represent ation	Sampling	Random selection	Non response bias	Data collection	Case Definition	Reliability and validity of study tool	Method of data collection	Prevalence period	Numerator and denominator	Summary Assessment
1	Tariku et al/2015.	Low risk	Low risk	Low risk	Low risk	Low risk	High risk	Low risk	Low risk	Low risk	Low risk	Low risk
2	Williams et al./2015/16.	Low risk	Low risk	Low risk	Low risk	Low risk	High risk	Low risk	Low risk	Low risk	Low risk	Low risk
3	Hailu et al/2015.	Low risk	Low risk	Low risk	Low risk	Low risk	Low risk	Low risk	Low risk	Low risk	Low risk	Low risk
4	Ssentongo et al/2016.	Low risk	Low risk	Low risk	Low risk	Low risk	High risk	Low risk	Low risk	Low risk	Low risk	Low risk
5	Abebe /2016.	High risk	High risk	Low risk	Low risk	Low risk	Low risk	High risk	Low risk	High risk	Low risk	Medium risk
6	Kangas et al /2016-2018.	Low risk	Low risk	Low risk	High risk	Low risk	Low risk	Low risk	Low risk	Low risk	Low risk	Low risk
7	Demissie et al/2006.	Low risk	Low risk	Low risk	Low risk	Low risk	Low risk	Low risk	Low risk	Low risk	Low risk	Low risk
8	Wirth et al/2013.	Low risk	Low risk	Low risk	Low risk	Low risk	Low risk	Low risk	Low risk	Low risk	Low risk	Low risk
9	Wirth et al/2019.	Low risk	Low risk	Low risk	High risk	Low risk	Low risk	Low risk	Low risk	Low risk	Low risk	Low risk
10	Ford et al/2015.	Low risk	Low risk	Low risk	Low risk	Low risk	Low risk	High risk	Low risk	low risk	Low risk	Low risk
11	Hune et al/2015-19.	High risk	Low risk	High risk	Low risk	Low risk	Low risk	Low risk	Low risk	Low risk	Low risk	Low risk
12	Rahman et al/2011/12.	Low risk	Low risk	Low risk	Low risk	Low risk	Low risk	High risk	Low risk	Low risk	Low risk	Low risk
13	Yisak et al/2019.	Low risk	Low risk	Low risk	Low risk	Low risk	Low risk	Low risk	Low risk	Low risk	Low risk	Low risk
14	Abrha et al/2014.	High risk	High risk	Low risk	Low risk	Low risk	Low risk	Low risk	Low risk	Low risk	Low risk	Low risk
15	Christine et al/2009.	High risk	High risk	Low risk	Low risk	Low risk	Low risk	Low risk	Low risk	Low risk	Low risk	Low risk
16	UNICEF/2012.	High risk	High risk	Low risk	Low risk	Low risk	Low risk	Low risk	Low risk	Low risk	Low risk	Low risk
17	Kosal et al/2014.	High risk	High risk	High risk	Low risk	Low risk	Low risk	Low risk	Low risk	Low risk	Low risk	Low risk
18	Orsango et al/2017.	High risk	Low risk	Low risk	Low risk	Low risk	Low risk	Low risk	Low risk	Low risk	Low risk	Low risk
19	Omer et al/2017-19.	Low risk	Low risk	Low risk	Low risk	Low risk	High risk	Low risk	Low risk	Low risk	Low risk	Low risk
20	Mbunga et al/2019.	Low risk	Low risk	Low risk	Low risk	Low risk	Low risk	Low risk	Low risk	Low risk	Low risk	Low risk
21	Andersen et al/2019.	Low risk	Low risk	Low risk	Low risk	Low risk	Low risk	Low risk	Low risk	low risk	Low risk	Low risk
22	Bahizire et al/2013.	Low risk	Low risk	Low risk	Low risk	Low risk	Low risk	Low risk	Low risk	Low risk	Low risk	Low risk
23	Fançony et al/2015.	Low risk	Low risk	Low risk	Low risk	Low risk	Low risk	Low risk	Low risk	Low risk	Low risk	Low risk
24	Harvey-Leeson et al/2014.	Low risk	Low risk	Low risk	Low risk	Low risk	Low risk	Low risk	Low risk	Low risk	Low risk	Low risk
25	Danquah et al/2010.	Low risk	Low risk	Low risk	Low risk	Low risk	Low risk	Low risk	Low risk	Low risk	Low risk	Low risk
26	Gashu et al/2011/12.	High risk	Low risk	Low risk	Low risk	Low risk	Low risk	Low risk	Low risk	Low risk	Low risk	Low risk
27	Wirth et al/2013.	Low risk	Low risk	Low risk	Low risk	Low risk	Low risk	Low risk	Low risk	Low risk	Low risk	Low risk
28	Wirth et al/2019.	Low risk	Low risk	Low risk	Low risk	Low risk	Low risk	Low risk	Low risk	Low risk	Low risk	Low risk
29	Kessy et al/2014/15.	High risk	Low risk	Low risk	Low risk	Low risk	Low risk	Low risk	Low risk	low risk	Low risk	Low risk
30	Kikafunda et al/2008.	High risk	High risk	Low risk	High risk	Low risk	Low risk	High risk	Low risk	Low risk	Low risk	Medium risk
31	Roba et al/2014.	High risk	High risk	Low risk	Low risk	Low risk	Low risk	Low risk	Low risk	Low risk	Low risk	Low risk
32	Randrianarisoa et al/2016-2018.	High risk	Low risk	Low risk	Low risk	Low risk	High risk	Low risk	Low risk	Low risk	Low risk	Low risk
33	Ford et al/2015.	Low risk	Low risk	Low risk	Low risk	Low risk	Low risk	Low risk	Low risk	Low risk	Low risk	Low risk
34	Msaki et al/2015/16.	High risk	High risk	Low risk	Low risk	Low risk	Low risk	Low risk	Low risk	Low risk	Low risk	Low risk
35	Simbauranga et al/2012/13.	High risk	Low risk	Low risk	High risk	Low risk	High risk	Low risk	Low risk	Low risk	Low risk	Medium risk
36	Bahati et al/2017.	Low risk	Low risk	Low risk	Low risk	Low risk	Low risk	Low risk	Low risk	low risk	Low risk	Low risk
37	Swareldhab et al/2019.	Low risk	Low risk	Low risk	Low risk	Low risk	Low risk	Low risk	Low risk	low risk	Low risk	Low risk
38	UNICEF/2013.	Low risk	Low risk	High risk	High risk	Low risk	Low risk	Low risk	Low risk	Low risk	Low risk	Low risk
39	Hoque et al/2010.	Low risk	Low risk	High risk	Low risk	Low risk	High risk	Low risk	Low risk	Low risk	Low risk	Low risk
40	Gashu et al/2011/12.	Low risk	Low risk	Low risk	Low risk	Low risk	High risk	Low risk	Low risk	Low risk	Low risk	Low risk
41	Ferede et al/2018.	Low risk	Low risk	Low risk	Low risk	Low risk	Low risk	Low risk	Low risk	Low risk	Low risk	Low risk
42	Hess et al/2010.	Low risk	Low risk	Low risk	Low risk	High risk	Low risk	Low risk	Low risk	Low risk	Low risk	Low risk
43	Harun-Or-Rashid et al/2005.	Low risk	Low risk	Low risk	Low risk	Low risk	Low risk	Low risk	Low risk	Low risk	Low risk	Low risk
44	Laillou et al/2014.	Low risk	Low risk	Low risk	Low risk	Low risk	Low risk	Low risk	Low risk	Low risk	Low risk	Low risk
45	Atukunda et al/2015	Low risk	Low risk	Low risk	Low risk	Low risk	Low risk	Low risk	Low risk	Low risk	Low risk	Low risk
46	Kosal et al/2014.	Low risk	Low risk	Low risk	Low risk	Low risk	Low risk	Low risk	Low risk	Low risk	Low risk	Low risk
47	Melku et al/2015.	Low risk	Low risk	Low risk	Low risk	Low risk	High risk	Low risk	Low risk	Low risk	Low risk	Low risk
48	Kuziga et al/2014.	High risk	Low risk	Low risk	High risk	High risk	High risk	High risk	Low risk	High risk	Low risk	High risk
49	Gebreegziabiher et al/2013.	High risk	Low risk	Low risk	High risk	Low risk	Low risk	Low risk	Low risk	Low risk	Low risk	Low risk
50	Tekile et al/2016.	Low risk	Low risk	Low risk	Low risk	Low risk	Low risk	Low risk	Low risk	Low risk	Low risk	Low risk
51	Molla et al/2018.	Low risk	Low risk	Low risk	Low risk	Low risk	High risk	Low risk	Low risk	Low risk	Low risk	Low risk
52	Orsango et al/2017.	High risk	Low risk	Low risk	Low risk	Low risk	Low risk	Low risk	Low risk	Low risk	Low risk	Low risk
53	Woldegebriel et al/2016.	High risk	Low risk	Low risk	High risk	Low risk	High risk	Low risk	Low risk	Low risk	Low risk	Medium risk
54	Malako et al/2017.	Low risk	Low risk	High risk	Low risk	Low risk	Low risk	High risk	High risk	low risk	Low risk	Medium risk
55	Woldie et al/2014.	Low risk	Low risk	High risk	Low risk	Low risk	Low risk	High risk	High risk	low risk	Low risk	Medium risk
56	Tegegne et al/2021.	Low risk	Low risk	Low risk	Low risk	Low risk	High risk	Low risk	Low risk	Low risk	Low risk	Low risk
57	Jembere et al/2019.	High risk	Low risk	Low risk	Low risk	Low risk	High risk	Low risk	Low risk	Low risk	Low risk	Low risk
58	Mohammed et al/2016.	Low risk	Low risk	Low risk	Low risk	Low risk	High risk	High risk	Low risk	Low risk	Low risk	Low risk
59	Gari et al/2014.	Low risk	Low risk	Low risk	Low risk	Low risk	Low risk	Low risk	Low risk	Low risk	Low risk	Low risk
60	Gari et al/2015.	Low risk	Low risk	Low risk	Low risk	High risk	Low risk	High risk	Low risk	Low risk	Low risk	Low risk
61	Adugna et al/2019/20	Low risk	Low risk	Low risk	Low risk	Low risk	Low risk	Low risk	Low risk	Low risk	Low risk	Low risk
62	Mollah et al/2017.	High risk	High risk	Low risk	Low risk	Low risk	Low risk	Low risk	Low risk	Low risk	Low risk	Low risk
63	Asresie et al/2016.	Low risk	Low risk	Low risk	Low risk	Low risk	Low risk	Low risk	Low risk	Low risk	Low risk	Low risk
64	Shrestha, Neeta/2017.	High risk	High risk	Low risk	Low risk	Low risk	Low risk	Low risk	Low risk	Low risk	Low risk	Low risk
65	Keokenchanh et al/2017.	Low risk	Low risk	Low risk	Low risk	High risk	Low risk	High risk	Low risk	High risk	Low risk	Medium risk
66	Mboya et al/2016.	Low risk	Low risk	Low risk	Low risk	Low risk	Low risk	Low risk	Low risk	Low risk	Low risk	Low risk
67	Rahman et al/2011.	Low risk	Low risk	Low risk	Low risk	Low risk	High risk	Low risk	Low risk	Low risk	Low risk	Low risk
68	Palacios et al/2012.	Low risk	Low risk	Low risk	Low risk	Low risk	Low risk	Low risk	Low risk	Low risk	Low risk	Low risk
69	Islam, GM Rabiul/2011.	Low risk	Low risk	Low risk	Low risk	Low risk	Low risk	Low risk	Low risk	Low risk	Low risk	Low risk
70	Mollah et al/2018/19.	High risk	Low risk	Low risk	High risk	Low risk	High risk	Low risk	Low risk	Low risk	Low risk	Medium risk
71	Afroja et al/2011.	Low risk	Low risk	Low risk	Low risk	High risk	Low risk	Low risk	Low risk	Low risk	Low risk	Low risk

Risk of bias assessment tool: Yes (low risk); No (high risk)

1. Representation: Was the study population a close representation of the national population?
2. Sampling: Was the sampling frame a true or close representation of the target population?
3. Random selection: Was some form of random selection used to select the sample OR was a census undertaken?
4. Non-response bias: Was the likelihood of non-response bias minimal?
5. Data collection: Were data collected directly from the subjects?
6. Case definition: Was an acceptable case definition used in the study?
7. Reliability and validity of study tool: Was the study instrument that measured the parameter of interest show to have reliability and validity?
8. Data collection: Was the same mode of data collection used for all subjects?
9. Prevalence period: Was the length of the prevalence period for the parameter of interest appropriate?
10. Numerators and denominators: Were the numerator(s) and denominator(s) for the parameter of interest appropriate?

The overall risk of bias scored based on the number of high risk of bias per study: low risk (≤2), moderate risk (3–4), and high risk (≥5).
