# Supplementary material for: Prevalence and Determinants of Stunting-Anemia and Wasting-Anemia Comorbidities and Micronutrient Deficiencies in Children Under 5 in the Least-Developed Countries: A Systematic Review and Meta-analysis
Source: Nutr Rev. 2024 May 31;83(2):e178–94. doi: 10.1093/nutrit/nuae063 (PMC11723162; doi:10.1093/nutrit/nuae063)
Supplement: nuae063_Supplementary_Data [file nuae063_supplementary_data.zip › nuae063_Supplementary_Data/S5 Supportive document -Table 1-4.docx]

**S5 Supportive document - Table 1:** Characteristics of included studies for prevalence of vitamin A deficiency among under-five children in least developed countries, 2006-2019.

| ID | Authors | Year of study | Study design | Country | Age in Month | Diagnosis method | Cut point | Sample | Prevalence of Vitamin A deficiency |
| --- | --- | --- | --- | --- | --- | --- | --- | --- | --- |
| 1 | Tariku et al^52^. | 2015 | Cross-sectional | Ethiopia | 24-59months | Clinically | N/A | 681 | 8.66 |
| 2 | Williams et al ^53^. | 2015/16 | Cross-sectional | Malawi | 6-59 months | Ultraviolet excitation fluorescence | <0.70 μmol/L | 1102 | 3.6 |
| 3 | Hailu et al ^56^. | 2015 | Cross-sectional | Ethiopia | 6-59 months | High performance liquid chromatography (HPLC) | <0.70 μmol/L | 4589 | 13.9 |
| 4 | Yisak et al ^54^. | 2019 | Cross-sectional | Ethiopia | 12-50 months | Clinically | N/A | 581 | 2 |
| 5 | Ssentongo et al ^63^. | 2016 | Cross-sectional | Uganda | 6-59 months | Retinol binding protein enzyme immunoassay | 0.825 μmol/L. | 424 | 8.9 |
| 6 | Abebe ^57^. | 2016 | Cross-sectional | Ethiopia | 36-59 months | High performance liquid chromatography (HPLC) | <0.70 μmol/L | 204 | 32.7 |
| 7 | Kangas et al ^61^. | 2016-2018 | Cross-sectional | Burkina Faso | 6-59 months | Serum concentrations of retinol binding protein (RBP) | <0.70 μmol/L | 801 | 9 |
| 8 | Demissie et al ^58^. | 2006 | Cross-sectional | Ethiopia | 6-48 months | High performance liquid chromatography (HPLC) | <0.70 μmol/L | 568 | 34 |
| 9 | Wirth et al ^64^. | 2013 | Cross-sectional | Sierra Leone | 6-59 months | Retinol binding protein enzyme immunoassay | <0.70 μmol/L | 654 | 17.4 |
| 10 | Wirth et al ^65^. | 2019 | Cross-sectional | Somalia | 6-59 months | Enzyme-linked immunosorbent assay | <0.70 μmol/L | 1480 | 34.4 |
| 11 | Ford et al ^59^. | 2015 | Cross-sectional | Uganda | 12-23 Months | High performance liquid chromatography (HPLC) | <0.70 μmol/L | 1260 | 5 |
| 12 | Hune et al ^60^. | 2015/19 | Cross-sectional | Ethiopia | 6–59 months | Clinically | N/A | 267 | 7.8 |
| 13 | Abrha et al ^41^. | 2014 | Cross-sectional | Ethiopia | 24-59 months | Clinically | N/A | 1230 | 1.5 |
| 14 | Rahman et al ^62^. | 2011/12 | Cross-sectional | Bangladeshi | 6–59 months | High performance liquid chromatography (HPLC) | <0.70 μmol/L | 873 | 20.5 |
| 15 | Christine et al^55^. | 2009 | Cross-sectional | Zambia | 24-59 months | Pye Unicam Philips system | <0.70 μmol/L | 664 | 56 |
| 16 | UNICEF^48^. | 2012 | Cross-sectional | Bangladeshi | 6–59 months | Retinol binding protein enzyme immunoassay | <0.70 μmol/L | 1200 | 20.5 |
| 17 | Kosal et al^47^. | 2014 | Cross-sectional | Cambodia | 6–59 months | Not specified | <0.70 μmol/L | 1000 | 3.2 |

**S5 Supportive document - Table 2:** Characteristics of included studies for prevalence of Iron deficiency anemia among under-five children in least developed countries, 2008-2019.

| ID | Authors | Year of study | Country | Study design | Age in Month | Diagnosis method | Cut point | Sample | Prevalence |
| --- | --- | --- | --- | --- | --- | --- | --- | --- | --- |
| 1 | Orsango et al ^71^. | 2017 | Ethiopia | Cross-sectional | 24-59 months | Not reported | Hb <11.0 g/dL and adjusted ferritin <12.0 μg/L | 331 | 25 |
| 2 | Omer et al ^83^. | 2017- 19 | Ethiopia | Retrospective study | 6-59 months | Red blood cell indices analysis | Combined low MCV, low MCH and high RDW (and Hgb of <11 g/dl for IDA) | 4,739 | 24.1 |
| 3 | Hailu AA ^56^. | 2015 | Ethiopia | Cross-sectional | 6-60 months | Immuneturbidimetriy method usingCobas6000 | Hb <11.0 g/dL and adjusted ferritin <12.0 μg/L | 1140 | 8.6 |
| 4 | Mbunga et al ^81^. | 2019 | Democratic Republic of the Congo | Cross-sectional | 12-60 months | Electrochemiluminescence immunoassay | Hb <11.0 g/dL and adjusted ferritin <12.0 μg/L | 432 | 7.5 |
| 5 | Andersen et al ^67^. | 2019 | Ethiopia | Cross-sectional | 6-60 months | Not reported | Hb <11.0 g/dL and adjusted ferritin <12.0 μg/L | 1162 | 25.3 |
| 6 | Bahizire et al ^77^. | 2013 | Democratic Republic of the Congo | Cross-sectional | 6-59 months | Enzyme-linked immunosorbent assay (ELISA) | Hb <11.0 g/dL and adjusted ferritin <12.0 μg/L | 833 | 7.44 |
| 7 | Fancony et al ^68^. | 2015 | Angola | Cross-sectional | 6-36 months | Automated autoanalizer | Hb <11.0 g/dL and adjusted ferritin <12.0 μg/L | 912 | 19.4 |
| 8 | Harvey-Leeson et al ^69^. | 2014 | Democratic Republic of the Congo | Cross-sectional | 6-59 months | Enzyme-linked immunosorbent assay (ELISA) | Hb <11.0 g/dL and adjusted ferritin <12.0 μg/L | 744 | 10 |
| 9 | Danquah et al ^78^. | 2010 | Rwanda | Cross-sectional | 6-59 months | Enzyme-linked immunosorbent assay (ELISA) | Hb <11.0 g/dL and adjusted ferritin <12.0 μg/L | 545 | 8.6 |
| 10 | Gashu et al ^79^. | 2011/12 | Ethiopia | Cross-sectional | 54–60 months | Electrochemilumenescense Immuno Assay | Hb <11.0 g/dL and adjusted ferritin <12.0 μg/L | 628 | 5.3 |
| 11 | Wirth et al ^64^. | 2013 | Sierra Leone | Cross-sectional | 6-59 months | Enzyme-linked immunosorbent assay (ELISA) | Hb <11.0 g/dL and adjusted ferritin <12.0 μg/L | 654 | 3.8 |
| 12 | Wirth et al ^65^. | 2019 | Somalia | Cross-sectional | 6-59 months | Enzyme-linked immunosorbent assay (ELISA) | Hb <11.0 g/dL and adjusted ferritin <12.0 μg/L | 1463 | 28.6 |
| 13 | Kessy et al ^80^. | 2014/15 | Tanzania | Cross-sectional | 2-59 months | Enzyme-linked immunosorbent assay (ELISA) | Hb <11.0 g/dL and adjusted ferritin <12.0 μg/L | 303 | 28.1 |
| 14 | Kikafunda et al ^66^. | 2008 | Uganda | Cross-sectional | 6-59 months | Not reported | WHO/United Nations University/UNICEF criteria but the exact number is not specified | 52 | 26.2 |
| 15 | Roba et al ^73^. | 2014 | Ethiopia | Cross-sectional | 6-23 Months | Enzyme-linked immunosorbent assay (ELISA) | Hb <11.0 g/dL and adjusted ferritin <15.0 μg/L | 162 | 29.6 |
| 16 | Randrianarisoa et al ^72^. | 2016-2018 | Madagascar | Cross-sectional | 24-59 months | Enzyme-linked immunosorbent assay (ELISA) | Hb <11.0 g/dL and adjusted ferritin <12.0 μg/L | 412 | 9.4 |
| 17 | Ford et al ^59^. | 2015 | Uganda | Cross-sectional | 12-23 Months | Enzyme-linked immunosorbent assay (ELISA) | Hb <11.0 g/dL and adjusted ferritin <12.0 μg/L | 1260 | 17.7 |
| 18 | Simbauranga et al ^84^. | 2012/13 | Tanzania | Cross-sectional | 6-59 months | Enzyme-linked immunosorbent assay (ELISA) | Hb <11.0 g/dL and adjusted ferritin <12.0 μg/L | 448 | 12.1 |
| 19 | Msaki et al ^82^. | 2015/16 | Tanzania | Cross-sectional | 6-59 months | Enzyme-linked immunosorbent assay (ELISA) | Hb <11.0 g/dL and adjusted ferritin <12.0 μg/L | 8014 | 58.8 |
| 20 | Bahati et al ^76^. | 2017 | Democratic Republic of Congo | Cross-sectional | 6-59 months | Enzyme-linked immunosorbent assay (ELISA) | Hb <11.0 g/dL and adjusted ferritin <12.0 μg/L | 1088 | 31 |
| 21 | Swareldhab et al ^74^. | 2019 | Sudan | Cross-sectional | 6-59 months | Not reported | Hb <11.0 g/dL and adjusted ferritin <12.0 μg/L | 9703 | 24 |
| 22 | UNICEF ^75^. | 2013 | Afghanistan | Cross-sectional | 6–59 months | Not reported | Ferritin levels <12ng/ml | 24348 | 13.7 |
| 23 | Hoque et al ^70^. | 2010 | Bangladesh | Cross-sectional | 6–59 months | Not reported | not reported | 331 | 19 |

**S5 Supportive document - Table 3:** Characteristics of included studies for prevalence of iodine deficiency among under-five children in least developed countries, 2005-2018.

| ID | Authors | Year of study | Country | Study Design | Age in Month | Diagnosis method | Cut point | Sample | Prevalence |
| --- | --- | --- | --- | --- | --- | --- | --- | --- | --- |
| 1 | Gashu et al ^86^. | 2011/12 | Ethiopia | Cross-sectional | 54–60 months | Electro chemilumen escense immunoassay | <100 μg/l | 449 | 86.6 |
| 2 | Ferede et al ^85^. | 2018 | Ethiopia | Cross-sectional | 6–59 months | Not specified | <100 μg/l | 812 | 11.8 |
| 3 | Hess et al. | 2010 | Burkina Faso | RCT | 9 months | Ammonium persulfate methods | <100 μg/l | 661 | 14.8 |
| 4 | Harun-Or-Rashid et al ^87^. | 2005 | Bangladesh | Cross-sectional | 6-24 months | Iodometric titration method | <100 μg/l | 395 | 38.7 |
| 5 | Laillou et al ^88^. | 2014 | Cambodia | Cross-sectional | 6–59 months | Sandell–Kolt–koff method | <100 μg/l | 950 | 63.7 |
| 6 | Kosal et al ^47^. | 2014 | Cambodia | Cross-sectional | 6–59 months | Not specified | <100 μg/l | 1000 | 66 |
| 7 | Atukunda et al. | 2015 | Uganda | Follow up study | 20–24 months | Sandell–Kolt–koff method | <100 μg/l | 77 | 21.8 |

**S5 Supportive document - Table 4:** Characteristics of included studies to assess the comorbidity of anemia with stunting and wasting among under-five children in least developed countries, 2011-2021.

| ID | Authors | Year of study | Country | Study design | Age in Month | Diagnostic criteria for both malnutrition and anemia | Cut point to diagnose anemia | Sample | Prevalence |
| --- | --- | --- | --- | --- | --- | --- | --- | --- | --- |
| 1 | Randrianarisoa et al ^72^. | 2016-2018 | Madagascar | Cross-sectional | 24-59 months | WHO | Hb <11.0 g/dL | 414 | 14 |
| 2 | Melku et al ^102^. | 2015 | Ethiopia | Cross-sectional | 6-59 months | WHO | Hb <11.0 g/dL | 707 | 13.2 |
| 3 | Kuziga et al ^99^. | 2014 | Uganda | Cross-sectional | 6-59 months | WHO | Hb <11.0 g/dL | 476 | 18.4 |
| 4 | Gebreegziabiher et al ^95^. | 2013 | Ethiopia | Cross-sectional | 6-59 months | WHO | Hb <11.0 g/dL | 568 | 22.7 |
| 5 | Tekile et al ^111^. | 2016 | Ethiopia | Cross-sectional | 6-59 months | WHO | Hb <11.0 g/dL | 8279 | 25.44 |
| 6 | Molla et al ^104^. | 2018 | Ethiopia | Cross-sectional | 6-23 months | WHO | Hb <11.0 g/dL | 531 | 12.6 |
| 7 | Orsango et al ^89^. | 2017 | Ethiopia | Cross-sectional | 24-59 months | WHO | Hb <11.0 g/dL | 331 | 17.8 |
| 8 | Roba et al ^109^. | 2014 | Ethiopia | Cross-sectional | 6-23 months | WHO | Hb <11.0 g/dL | 215 | 25.1 |
| 9 | Woldegebriel et al ^112^. | 2016 | Ethiopia | Cross-sectional | 6-59 months | WHO | Hb <11.0 g/dL | 8155 | 21.7 |
| 10 | Malako et al ^100^. | 2017 | Ethiopia | Cross-sectional | 6-23 months | WHO | Hb <11.0 g/dL | 661 | 6.8 |
| 11 | Woldie et al ^113^. | 2014 | Ethiopia | Cross-sectional | 6-23 months | WHO | Hb <11.0 g/dL | 347 | 20.2 |
| 12 | Mbunga et al ^81^. | 2019 | Democratic Republic of Congo | Cross-sectional | 12-59 months | WHO | Hb <11.0 g/dL | 432 | 38.66 |
| 13 | Tegegne et al ^90^. | 2021 | Ethiopia | Cross-sectional | 6-23 months | WHO | Hb <11.0 g/dL | 770 | 20.5 |
| 14 | Jembere et al ^97^. | 2019 | Ethiopia | Cross-sectional | 6-59 months | WHO | Hb <11.0 g/dL | 413 | 18.64 |
| 15 | Mohammed et al ^103^. | 2016 | Ethiopia | Cross-sectional | 6-23 months | WHO | Hb <11.0 g/dL | 2902 | 23.9 |
| 16 | Gari et al ^94^. | 2014 | Ethiopia | Cross-sectional | 6-59 months | WHO | Hb <11.0 g/dL | 2984 | 15.7 |
| 17 | Gari et al ^94^. | 2015 | Ethiopia | Cross-sectional | 6-59 months | WHO | Hb <11.0 g/dL | 3128 | 20.5 |
| 18 | Adugna et al ^91^. | 2019/20 | Liberia | Cross-sectional | 6-59 months | WHO | Hb <11.0 g/dL | 2524 | 23.7 |
| 19 | Mollah et al ^105^. | 2017 | Bangladesh | Cross-sectional | 6-59 months | WHO | Hb <11.0 g/dL | 258 | 21.3 |
| 20 | Asresie et al ^93^. | 2016 | Ethiopia | Cross-sectional | 6-59 months | WHO | Hb <11.0 g/dL | 8462 | 25.4 |
| 21 | Shrestha, Neeta ^110^. | 2017 | Bhutan | Cross-sectional | 6-59 months | WHO | Hb <11.0 g/dL | 131 | 19.1 |
| 22 | Keokenchanh et al ^98^. | 2017 | Lao People’s Democratic Republic | Cross-sectional | 6-59 months | WHO | Hb <11.0 g/dL | 5086 | 15.5 |
| 23 | Mboya et al ^101^. | 2016 | Tanzania | Cross-sectional | 6-59 months | WHO | Hb <11.0 g/dL | 602 | 16.5 |
| 24 | Rahman et al ^108^. | 2011 | Bangladesh | Cross-sectional | 6-59 months | WHO | Hb <11.0 g/dL | 2234 | 23.4 |
| 25 | Palacios et al ^107^. | 2012 | Haiti | Cross-sectional | 6-59 months | WHO | Hb <11.0 g/dL | 897 | 16.6 |
| 26 | Islam, GM Rabiul ^96^. | 2011 | Bangladesh | Cross-sectional | 6-59 months | WHO | Hb <11.0 g/dL | 2068 | 22.3 |
| 27 | Mollah et al ^106^. | 2018/19 | Bangladesh | Cross-sectional | 6-59 months | WHO | Hb <11.0 g/dL | 258 | 21.3 |
| 28 | Afroja et al ^92^. | 2011 | Bangladesh | Cross-sectional | 6-59 months | WHO | Hb <11.0 g/dL | 2188 | 23.8 |
