# Supplementary material for: Prevalence and Determinants of Stunting-Anemia and Wasting-Anemia Comorbidities and Micronutrient Deficiencies in Children Under 5 in the Least-Developed Countries: A Systematic Review and Meta-analysis
Source: Nutr Rev. 2024 May 31;83(2):e178–94. doi: 10.1093/nutrit/nuae063 (PMC11723162; doi:10.1093/nutrit/nuae063)
Supplement: nuae063_Supplementary_Data [file nuae063_supplementary_data.zip › nuae063_Supplementary_Data/S6 Source of heterogeneity.docx]

**S6 Source of heterogeneity:** The source of heterogeneity for a meta-analysis assessing stunting-anemia, wasting-anemia comorbidities, and micronutrient deficiencies from 2005-2021.

| **Variables** | | **VAD** | | **Iron deficiency** | | **Iodine deficiency** | | **Stunting - Anemia** | | **Wasting- Anemia** | |
| --- | --- | --- | --- | --- | --- | --- | --- | --- | --- | --- | --- |
|  |  | **β** | **p-value** | **β** | **p-value** | **β** | **p-value** | **β** | **p-value** | **β** | **p-value** |
| Year of study | | -3.56 | 0.018 | 1.623 | 0.149 | -2.90 | 0.746 | -0.38 | 0.289 | 0.09 | 0.824 |
| Age | | 1.21 | 0.527 | 0.867 | 0.472 | -9.89 | 0.531 | 0.26 | 0.814 | 0.55 | 0.559 |
| Sample | | -0.001 | 0.732 | 0.498 | 0.400 | 0.06 | 0.390 | 0.01 | 0.092 | 0.01 | 0.752 |
| Regions | Asia | -8.80 | 0.532 | 13.59 | 0.315 |  |  | -4.85 | 0.353 | 3.06 | 0.049 |
|  | Eastern Africa | 6.37 | 0.593 | 15.02 | 0.105 |  |  | -6.39 | 0.198 | Reference | |
|  | Western Africa | Reference | | Reference | |  |  | Reference | |  |  |
|  | Middle Africa |  |  | 3.44 | 0.732 |  |  |  |  | 5.11 | 0.238 |
|  | Northeastern Africa |  |  | 2.17 | 0.895 |  |  |  |  |  |  |
|  | Caribbean |  |  |  |  |  |  | -9.5 | 0.172 |  |  |
