# Supplementary material for: Prevalence and Determinants of Stunting-Anemia and Wasting-Anemia Comorbidities and Micronutrient Deficiencies in Children Under 5 in the Least-Developed Countries: A Systematic Review and Meta-analysis
Source: Nutr Rev. 2024 May 31;83(2):e178–94. doi: 10.1093/nutrit/nuae063 (PMC11723162; doi:10.1093/nutrit/nuae063)
Supplement: nuae063_Supplementary_Data [file nuae063_supplementary_data.zip › nuae063_Supplementary_Data/S7 Meta funnel graphs.docx]

**S7 Meta funnel graphs:** Meta funnel presentation of the prevalence of stunting-anaemia and wasting-anaemia comorbidities with micronutrient deficiencies among children, 2000–2022, least developed countries.

**S5 Figure 1:** Meta funnel presentation of the prevalence of Vitamin A deficiency among children, 2006–2019, least developed countries.

**S5 Figure 2:** Meta funnel presentation of the prevalence of Iron deficiency anaemia among under five children, 2008-2019, least developed countries.

**S5 Figure 3:** Meta funnel presentation of the prevalence of Iodine deficiency among under five children, 2005–2018, least developed countries.

**S5 Figure 4:** Meta funnel presentation of the prevalence of stunting- anaemia comorbidity among under five children, 2011–2021, least developed countries.

**S5 Figure 5:** Meta funnel presentation of the prevalence of wasting- anaemia comorbidity among under five children, 2011–2019, least developed countries.
