# Supplementary material for: Prevalence and Determinants of Stunting-Anemia and Wasting-Anemia Comorbidities and Micronutrient Deficiencies in Children Under 5 in the Least-Developed Countries: A Systematic Review and Meta-analysis
Source: Nutr Rev. 2024 May 31;83(2):e178–94. doi: 10.1093/nutrit/nuae063 (PMC11723162; doi:10.1093/nutrit/nuae063)
Supplement: nuae063_Supplementary_Data [file nuae063_supplementary_data.zip › nuae063_Supplementary_Data/S8 sensitivity analysis.docx]

**Sensitivity analysis**

**S6 figure 1:** Result of sensitivity analysis of the 17 studies to assess vitamin A Deficiency among under five children in least developed countries.

**S6 figure 2:** Result of sensitivity analysis of the 23 studies to assess iron deficiency anaemia among under five children in least developed countries.

**S6 figure 3:** Result of sensitivity analysis of the 7 studies to assess iodine deficiency among under five children in least developed countries.

**S6 figure 4:** Result of sensitivity analysis of the 27 studies to assess stunting- anaemia comorbidity among under five children in least developed countries.

**S6 figure 5:** Result of sensitivity analysis of the 18 studies to assess wasting- anaemia comorbidity among under five children in least developed countries.
